# Harmonic Scale Factors of Fundamental Transitions for Dispersion‐corrected Quantum Chemical Methods

**DOI:** 10.1002/cphc.202400547

**Published:** 2024-10-28

**Authors:** Denis S. Tikhonov, Igor Gordiy, Danila A. Iakovlev, Alisa A. Gorislav, Mikhail A. Kalinin, Sergei A. Nikolenko, Ksenia M. Malaskeevich, Karina Yureva, Nikita A. Matsokin, Melanie Schnell

**Affiliations:** ^1^ Deutsches Elektronen-Synchrotron DESY Notkestr. 85 22607 Hamburg Germany; ^2^ Free Moscow University; ^3^ ChemU Corporation Ltd. 3106 Limassol 17 17 Gr. Xenopoulou St. Cyprus; ^4^ Independent researcher; ^5^ Organic Chemistry Department Institute of Chemistry Martin-Luther-University Halle-Wittenberg Kurt-Mothes-Straße 2 06120 Halle Germany; ^6^ Institute of Physical Chemistry Christian-Albrechts-Universität zu Kiel 24118 Kiel Germany

**Keywords:** DFT (density functional theory), Scale factors, Infrared spectroscopy, Molecular vibrations, Fitting, Dataset

## Abstract

This work provides a procedure and database for obtaining the vibrational frequency scale factors that align quantum chemically computed harmonic frequencies with experimental vibrational spectroscopic data. The database comprises 441 molecules of various sizes, from diatomics to the buckminsterfullerene C_60_. We provide scale factors for 27 dispersion‐corrected methods, 24 of which are DF‐D*n*/B with DF=BLYP, PBE, B3LYP, PBE0, D*n*=D3(BJ), D4, and B=6‐31G, def2‐SVP, def2‐TZVP, and three of them are the 3c‐family composite methods (HF‐3c, PBEh‐3c, and r^2^SCAN‐3c). The two scale factors are derived for each method: the absolute scaling, minimizing the absolute deviation of the scaled harmonic frequency from the experimental value, and the relative scaling, which minimizes an analogous relative deviation. The absolute type of scaling is recommended for frequencies above 2000 cm^−1^, while the relative scaling is optimal for frequencies below 2000 cm^−1^.

## Graphical abstract

This study presents two new sets of scaling factors for adjusting theoretical harmonic vibrational spectra computed using dispersion‐corrected density functional theory methods to better match experimental data. The first set (absolute scaling) better aligns with higher frequencies, while the second one (relative scaling) evenly reduces errors across the entire spectral range.

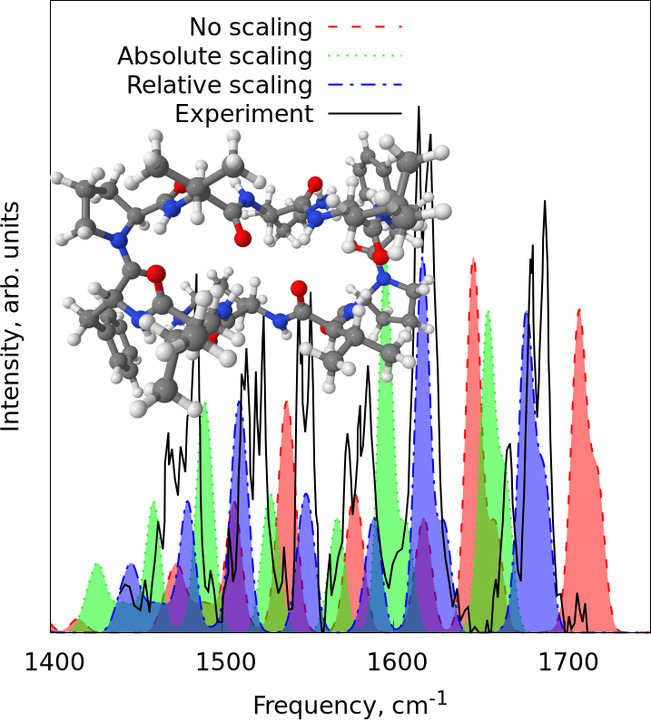

## 1 Introduction

Infrared (IR) spectroscopy is one of the important physicochemical methods in the chemists’ arsenal.^[1]^ This spectroscopic technique observes intra‐ and intermolecular vibrational motions, as their resonant excitation energies lie in the IR region, i. e., approximately from 100 to 10^4^ cm^−1^ (wavelengths from 0.1 mm to 780 nm).[^1^, ^2^] In addition to simple absorption/emission IR spectroscopy,^[3]^ there are other techniques, such as vibrational circular dichroism (VCD), which is sensitive to molecular chirality,[^4^, ^5^] vibrational Raman spectroscopy,^[6]^ inelastic neutron scattering (INS),[^6^, ^7^, ^8^] infrared/ultraviolet (IR‐UV) ion dip spectroscopy,^[9]^ infrared multiple photon dissociation (IRMPD) spectroscopy,[^10^, ^11^, ^12^, ^13^, ^14^] various messenger‐tagging methods (such as helium, nitrogen, or hydrogen molecular tag),[^15^, ^16^, ^17^, ^18^, ^19^, ^20^] that allow measuring spectra of ions, and vibrational sum frequency generation (VSFG) spectroscopy, that allows to probe molecular motions at the phase interfaces.[^21^, ^22^] All these vibrational spectroscopy methods allow us to inspect the properties of molecules, ions, and molecular aggregates in the gas, liquid, and solid phases and at the interfaces between phases. In addition to that, vibrational spectroscopic techniques are widely used in analytical chemistry.^[23]^ Even more, an important usage of IR spectroscopy includes astronomy and astrochemistry, with the existence of IR spectrometers on interplanetary probes and of various IR space telescopes, such as the recently retired Stratospheric Observatory For Infrared Astronomy (SOFIA)^[24]^ and recently launched James Webb Space Telescope (JWST).[^25^, ^26^]

However, interpreting the IR and other vibrational spectra can be complicated. Despite the existence of various vibrational diagnostic bands for molecular functional groups[^11^, ^12^, ^27^] and databases of the experimental IR spectra, such as the NIST Chemistry WebBook,^[28]^ most experimental studies of novel uncharacterized substances have to rely on quantum‐chemical calculations.[^5^, ^6^, ^8^, ^9^, ^16^, ^17^, ^18^, ^29^, ^30^, ^31^, ^32^, ^33^] To achieve a sufficiently good agreement of quantum‐chemical calculations and experiment requires the application of expensive computational methods and direct inclusion of anharmonic effects.[^6^, ^8^, ^34^, ^35^] However, such anharmonic calculations require significant expertise and computational efforts, which make them unfeasible for many practical cases.^[35]^ Therefore, the most widely used computational approach for the interpretation of experimental spectra is the harmonic frequency calculation with subsequent scaling of the resulting frequencies with a pre‐defined global scale factor.[^36^, ^37^, ^38^, ^39^, ^40^, ^41^] The scale factor accounts for two effects: 1) the quality of the quantum‐chemical approximation used and 2) anharmonic effects, which are, by definition, absent in the harmonic calculations.[^36^, ^42^] Therefore, updating the sets of scale factors, considering the expanding corpus of the experimentally determined vibrational frequencies and the new quantum‐chemical methods, is an interminable task. This effort is further facilitated by dedicated groups of researchers who diligently monitor and periodically incorporate new updates into these databases to ensure that the scale factors remain current and reflect the latest advances in the field.^[43]^

This work presents a new set of scale factors for 27 quantum‐chemical methods. In addition, these new scale factors have been rigorously tested and compared on several experimental data for structures to ensure their accuracy and reliability. The comprehensive examination of these scale factors across various classes of molecules enhances our understanding of their applicability and underscores their potential to advance computational chemistry methodology.

## Methods

### Dataset Selection

The information on the fundamental vibrational transitions of the molecules was extracted from the NIST Computational Chemistry Comparison and Benchmark Database (CCCBDB).^[44]^ The species and the gas‐phase fundamental transitions were chosen manually with the following guiding principles:

- Only neutral covalently bonded molecules in singlet ground states were selected.
- Only the most abundant isotopologues were taken.
- Experimental fundamental transitions for all normal modes should be assigned. Each diatomic molecule is characterized by a single vibrational number, while all *N*‐atomic molecules for N>2 (including linear) are characterized with 3N-6 fundamental modes. In the case of degenerate normal modes, every mode was given. The lowest energy vibration was disregarded for linear molecules, as this additional vibration usually corresponds to a doubly degenerate bending mode.

**Figure 1:**
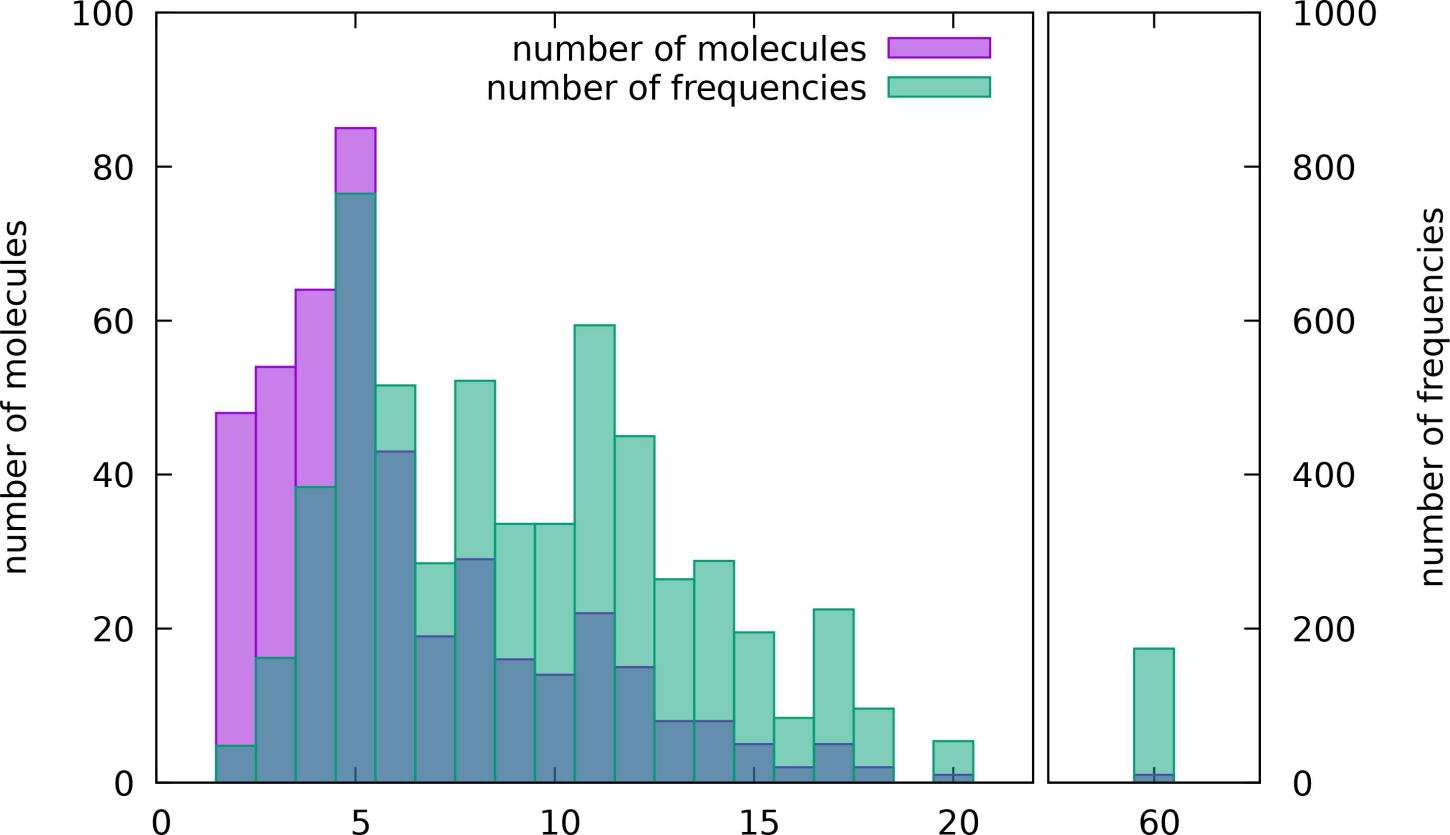
Number of molecules and corresponding number of vibrational frequencies comprised of a given number of atoms in the final dataset of fundamental frequencies. Note, that the two histograms have different *y*‐axes.

In addition to the molecules from the CCCBDB, nitrobenzene and uracil were added, with the full vibrational assignments given according to Refs. [45–47]. In total, the database contains 441 molecules of various sizes. The distribution of molecular sizes is given in Figure 1. The neutral closed‐shell species were chosen for the training set to unify the input files used to calculate the IR spectra.

The experimental and theoretical frequencies for each molecule of the dataset were ordered by their values. Naturally, such a crude procedure creates some misassignments, as some of the vibrational modes in calculations can have different energy ordering compared to the experiment. Nevertheless, such an approach is formalizable and scalable, and the effect of such misassignments should be compensated in large datasets.

### Quantum‐chemical Calculations

The first set of 24 methods can be written as “DF‐D*n*/B”. Here, “DF” denotes a correlation‐exchange density functional, “D*n*” is a dispersion correction, and “B” is the basis set used. For DF, we chose two pure generalized gradient approximation (GGA) functionals (BLYP[^48^, ^49^] and PBE^[50]^) and their popular hybrid counterparts (B3LYP,^[51]^ PBE0^[52]^). As dispersion corrections (D*n*), we considered D3(BJ)^[53]^ and the newly developed D4^[54]^ corrections. As the basis sets (B), we used a small 6–31G basis set[^55^, ^56^] and two Karsruhe‐type basis sets,^[57]^ def2‐SVP and def2‐TZVP. The second set of three methods includes three examples of 3c‐composite methods, namely HF‐3c,^[58]^ PBEh‐3c,^[59]^ and r^2^SCAN‐3c.^[60]^ The harmonic vibrational frequencies of the most abundant isotopologues after the geometry optimization at the corresponding level of theory were done using the ORCA 5 quantum‐chemical software.[^61^, ^62^] With elements of the fifth row and beyond, the automatically chosen by ORCA effective core potentials def2‐ECP for these elements were applied.[^57^, ^63^, ^64^] For potentially conformationally flexible molecules, an initial conformational search was performed at the GFN2‐xTB^[65]^ level of theory using the CREST[^66^, ^67^] software. Further optimization and harmonic frequency calculations were done for the lowest‐energy conformer found.

**Figure 2:**
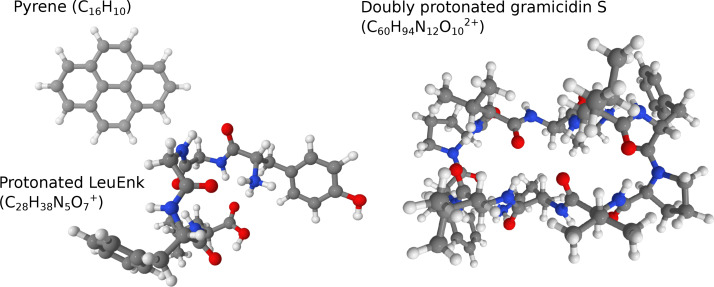
Structures and chemical formulas of three molecules that were used to illustrate the performance of the scale factors obtained in this work. The colors of spheres denote the atom type: white denotes hydrogens, gray denotes carbons, blue denotes nitrogens, and red denotes oxygens.

The calculations were performed in the same fashion but with a few minor differences for the illustrative molecular systems (pyrene, protonated LeuEnk, and doubly protonated gramicidin S, see Figure 2). For pyrene, in addition to harmonic spectra at all 27 chosen methods, the intensities of the overtones and combination bands at the r^2^SCAN‐3c level of theory were computed with the “NearIR” procedure of ORCA.^[68]^ For protonated LeuEnk, only GGA and 3c‐methods were tested. Nine representative conformers, given in Ref. [33], were optimized at the corresponding level of theory, and then their IR harmonic spectra were averaged with the weights provided in Ref. [33]. We did not repeat the extensive work on the conformational search for the LeuEnk, relying on the results from the corresponding work.^[33]^ For the doubly protonated gramicidin S, the given conformer from Ref. [30] was used after re‐optimization at several levels of theory from our list of methods. The IR spectra for these three systems were convoluted with the Gaussian functions with full width at half‐maximum FWHM=30 cm^−1^ for pyrene, FWHM=110 cm^−1^ for protonated LeuEnk, and FWHM=10 cm^−1^ for gramicidin S, to match the widths observed in the experimental spectra.

### Least‐squares Fitting of the Scale Factors

The goal of the optimization of the scale factors *s* was to adjust every quantum‐chemically computed harmonic frequency $\nu^{\left(\mathrm{calc}\right)}$ at a given level of theory to the experimental fundamental transition frequency $\nu^{\left(\exp\right)}$ as[^38^, ^41^]

(1) $$s\cdot \nu^{\left(\mathrm{calc}\right)}=\nu^{\left(\exp\right)}.$$

To find the best scale factors that approximately fulfill the requirement given in Equation 1, two types of least‐squares (LSQ) fitting were applied, which minimized a given root‐mean‐square deviation (RMSD). The first type of scale factor that we obtained is denoted as the absolute scale factor (*s*_abs_). The second type of scale factor is called the relative scale factor (*s*_rel_).

The scale factor *s*_abs_ was obtained by minimizing the following absolute RMSD (aRMSD):[^38^, ^41^]

(2) $${\mathrm{aRMSD}}^{2}=\frac{1}{N_{\mathrm{tot}}}\sum_{m=1}^{M}\sum_{k=1}^{K_{m}}{\left(s_{\mathrm{abs}}\cdot \nu_{mk}^{\left(\mathrm{calc}\right)}-\nu_{mk}^{\left(\exp\right)}\right)}^{2}\to \min,$$

where *m* enumerates *M* molecules in the training set, *k* enumerates the computed and experimental frequencies for the *m*‐th molecule, and $N_{\mathrm{tot}}=\sum_{m=1}^{M}K_{m}$ is the total number of frequencies in the training set. The *s*_rel_ was obtained by minimizing a relative RMSD (rRMSD):

(3) $${\mathrm{rRMSD}}^{2}=\frac{1}{N_{\mathrm{tot}}}\sum_{m=1}^{M}\sum_{k=1}^{K_{m}}{\left(s_{\mathrm{rel}}\cdot \frac{\nu_{mk}^{\left(\mathrm{calc}\right)}}{\nu_{mk}^{\left(\exp\right)}}-1\right)}^{2}\to \min.$$

The optimal values of the *s*_abs_ and *s*_rel_ and their fit uncertainties $\sigma_{\mathrm{abs}}$ and $\sigma_{\mathrm{rel}}$ according to Equations 2 and 3 are given as[^38^, ^41^]

(4) $$s_{\mathrm{abs}}\pm \sigma_{\mathrm{abs}}=\frac{\sum_{m=1}^{M}\sum_{k=1}^{K_{m}}\nu_{mk}^{\left(\mathrm{calc}\right)}\nu_{mk}^{\left(\exp\right)}}{\sum_{m=1}^{M}\sum_{k=1}^{K_{m}}{\left(\nu_{mk}^{\left(\mathrm{calc}\right)}\right)}^{2}}\pm \frac{{\mathrm{aRMSD}}_{\min}}{\sqrt{\sum_{m=1}^{M}\sum_{k=1}^{K_{m}}{\left(\nu_{mk}^{\left(\mathrm{calc}\right)}\right)}^{2}}}$$

and

(5) $$s_{\mathrm{rel}}\pm \sigma_{\mathrm{rel}}=\frac{\sum_{m=1}^{M}\sum_{k=1}^{K_{m}}\nu_{mk}^{\left(\mathrm{calc}\right)}/\nu_{mk}^{\left(\exp\right)}}{\sum_{m=1}^{M}\sum_{k=1}^{K_{m}}{\left(\nu_{mk}^{\left(\mathrm{calc}\right)}/\nu_{mk}^{\left(\exp\right)}\right)}^{2}}\;\;\pm \frac{{\mathrm{rRMSD}}_{\min}}{\sqrt{\sum_{m=1}^{M}\sum_{k=1}^{K_{m}}{\left(\nu_{mk}^{\left(\mathrm{calc}\right)}/\nu_{mk}^{\left(\exp\right)}\right)}^{2}}},$$

where ${\mathrm{aRMSD}}_{\min}$ and ${\mathrm{rRMSD}}_{\min}$ denote minimal values of Equations 2 and 3, obtained with optimal *s*_abs_ and *s*_rel_ according to Equations 4 and 5, respectively.

## 2 Results and Discussion

The resulting scaling factors and corresponding aRMSD values obtained by the procedure described in Section 2 are given in Table 1. The aRMSD values can be thought of as the expected accuracy of the given combination of the method and the scale factor, i. e., the deviations of the scaled spectra from the experimental ones are expected to be within the ±aRMSD interval. There are a few trends that can be observed. First of all, $s_{\mathrm{rel}}\geq s_{\mathrm{abs}}$ for each given method. Secondly, the scale factors for the DF−D3(BJ)/B methods are within accuracy compared to DF−D4/B, suggesting that the effects of D3(BJ) and D4 corrections are similar. The last trend observed is a systematic decrease of the aRMSD values upon improving the basis set quality, namely aRMSD(DF‐D*n*/def2‐TZVP)≤aRMSD(DF‐D*n*/def2‐SVP)≤aRMSD(DF‐D*n*/6‐31G).

**Table 1:** Optimal absolute (*s*_abs_) and relative (*s*_rel_) harmonic frequency scaling factors were determined for the listed quantum‐chemical approximations with Equations 4 and 5, respectively. aRMSD denotes the values of the absolute root‐mean‐square deviations (Equation 2) computed for the dataset sets of frequencies with the corresponding scale factors.

	Method		Absolute scaling		Relative scaling	
DF	D*n*	Basis	*s*_abs_	aRMSD, cm^−1^	*s*_rel_	aRMSD, cm^−1^
		6‐31G	0.9911(6)	72	1.018(2)	84
	D3BJ	def2‐SVP	0.9972(3)	41	1.013(1)	48
BLYP		def2‐TZVP	0.9960(3)	41	1.017(1)	52
		6‐31G	0.9912(6)	72	1.017(2)	83
	D4	def2‐SVP	0.9973(3)	41	1.013(1)	48
		def2‐TZVP	0.9961(3)	41	1.017(1)	52
		6‐31G	0.9848(6)	67	1.010(2)	79
	D3BJ	def2‐SVP	0.9904(4)	42	1.006(1)	48
PBE		def2‐TZVP	0.9923(3)	39	1.011(1)	48
		6‐31G	0.9849(6)	67	1.010(2)	78
	D4	def2‐SVP	0.9905(4)	42	1.005(1)	48
		def2‐TZVP	0.9925(3)	39	1.010(1)	47
		6‐31G	0.9611(5)	63	0.981(1)	71
	D3BJ	def2‐SVP	0.9664(3)	36	0.975(1)	39
B3LYP		def2‐TZVP	0.9671(3)	32	0.979(1)	37
		6‐31G	0.9613(5)	63	0.981(1)	72
	D4	def2‐SVP	0.9666(3)	36	0.976(1)	39
		def2‐TZVP	0.9673(3)	32	0.979(1)	37
		6‐31G	0.9509(5)	59	0.969(1)	66
	D3BJ	def2‐SVP	0.9555(3)	39	0.963(1)	41
PBE0		def2‐TZVP	0.9591(3)	33	0.967(1)	36
		6‐31G	0.9510(5)	59	0.969(1)	66
	D4	def2‐SVP	0.9556(3)	39	0.963(1)	41
		def2‐TZVP	0.9591(3)	33	0.967(1)	36
HF‐3c			0.8400(6)	82	0.858(1)	89
PBEh‐3c			0.9329(3)	33	0.935(1)	33
r^2^SCAN‐3c			0.9688(3)	35	0.982(1)	41

In order to illustrate the applicability of the obtained scale factors, we performed calculations for three molecular systems of various sizes and complexity for which experimental vibrational spectra are available from the literature (Figure 2). Here, we will demonstrate only the results at the r^2^SCAN‐3c level of theory, and all the other comparisons can be found in ESI.

**Figure 3:**
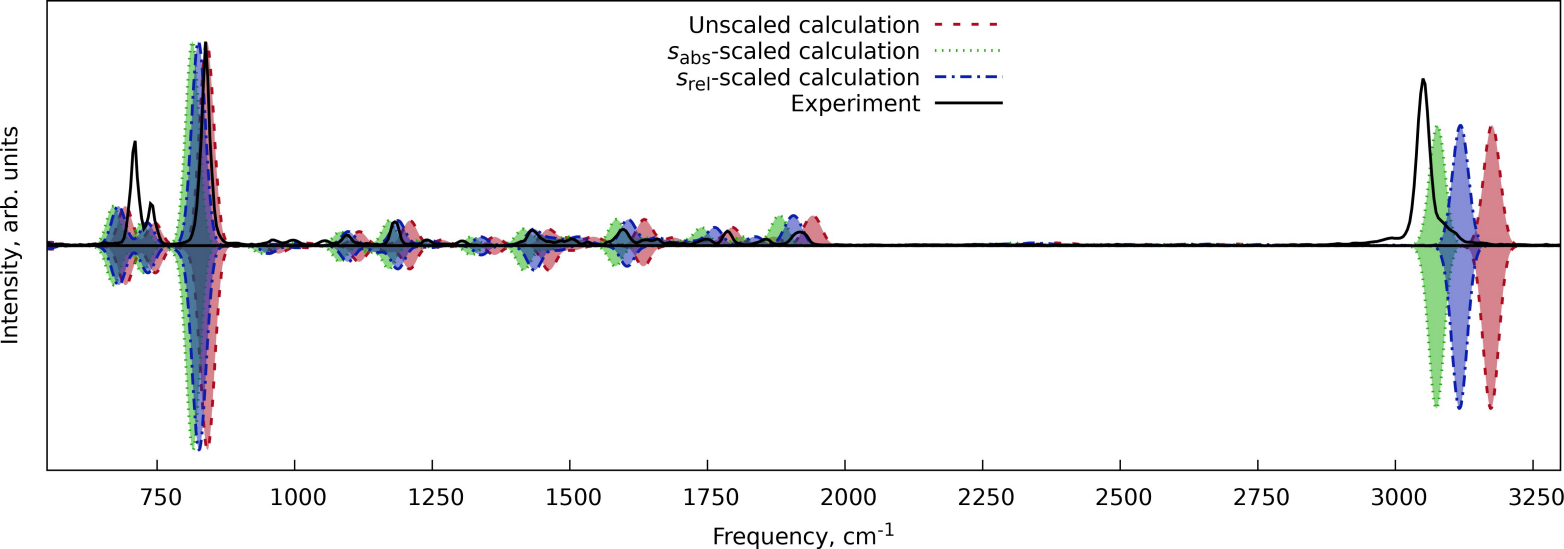
Comparison of the scaled and unscaled harmonic IR spectrum of pyrene at the r^2^SCAN‐3c level of theory with the experimental data from NIST Chemistry WebBook.^[28]^ The top theoretical trace shows a spectrum with the harmonic fundamentals, overtones, and combination bands, while the lower trace shows the harmonic spectrum with fundamental transitions only.

The first system was pyrene (C_16_H_10_), a four‐ring polycyclic aromatic hydrocarbon, for which the experimental gas‐phase IR spectra were available on the NIST Chemistry WebBook.^[28]^ The comparison of the harmonic spectra with fundamentals only and with overtones and combination bands is shown in Figure 3. We can see that the same scale factors can be used to adjust the positions of the overtones and combination bands while no anharmonic corrections are applied. The *s*_abs_ demonstrates the best performance for the C−H stretching vibrations (above 2000 cm^−1^), while the *s*_rel_ performs best at the fingerprint region below 2000 cm^−1^, accounting for a generally better representation of the spectral features.

**Figure 4:**
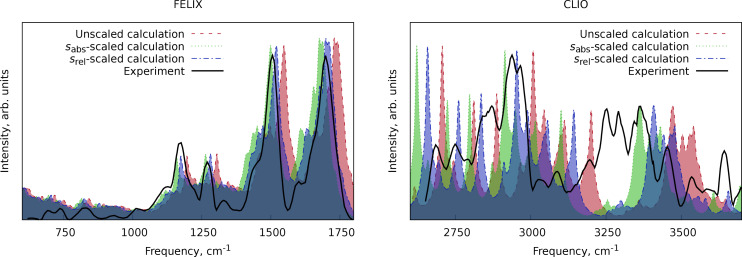
Comparison of the scaled and unscaled harmonic IR spectrum of the closed form of the protonated LeuEnk polypeptide at the r_2_SCAN‐3c level of theory with the experimental data from Ref. [33]. The numerical experimental data were extracted from the manuscript images using WebPlotDigitizer software.^[69]^

A similar pattern can be observed for the protonated version of the LeuEnk polypeptide (C_28_H_38_N_5_O_7_^+^) in the spectra in the X−H stretching region (X=C, N, O) taken with the CLIO setup^[70]^ and in the spectra in the fingerprint region taken at the FELIX facility^[71]^ (Figure 4). The *s*_rel_‐scaled spectrum best fits to the lower energy spectral region compared to the *s*_abs_‐scaled one. The X−H stretching region has a lot of bands, probably arising from a complicated conformational composition, resonances, and overtones, which makes this region more difficult to analyze. Nevertheless, for the most visible bands in the harmonic calculations, the *s*_abs_‐scaled spectrum better matches in peak positions than the *s*_rel_‐scaled one.

**Figure 5:**
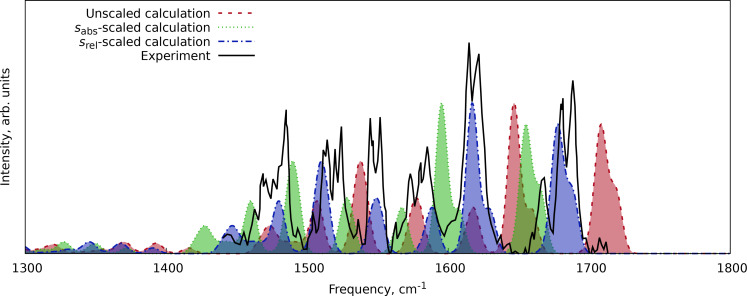
Comparison of the scaled and unscaled harmonic IR spectrum of double protonated conformer A of gramicidin S at the r^2^SCAN‐3c level of theory with the experimental data from Ref. [30]. The numerical experimental data were extracted from the manuscript images using WebPlotDigitizer software.^[69]^

Our last example was the double protonated conformer A of gramicidin S (C_60_H_94_N_12_O_10_^2+^), for which the fingerprint region helium‐tagging action spectrum was available.^[30]^ Here (Figure 5), we observe the same trend as in the previous examples (Figures 3 and 4): the *s*_rel_‐scaled spectrum shows a good agreement with peak positions, while the *s*_abs_‐scaled spectrum overestimates the red shift of the peak positions.

## 3 Conclusions

Here, we presented the dataset and the procedure for obtaining scale factors for the IR vibrational spectra. We developed two sets of scale factors, derived from two different principles: absolute scaling (*s*_abs_), by taking into account absolute deviations of the theoretical frequency from the experimental ones, and relative scaling (*s*_rel_), which minimizes the relative deviation of one frequency from another. The numerical demonstrations with three molecular systems of various sizes (pyrene and two ionic peptides: protonated LeuEnk and doubly protonated gramicidin S) show that the *s*_abs_ provides better results for the hydrogen‐stretching region (above 2000 cm^−1^), while the *s*_rel_ outperforms *s*_abs_ in the fingerprint region (below 2000 cm^−1^). Thus, we recommend joint usage of these scale factors to improve the predictions for different spectral regions. The RMSD values accompanying these scale factors can be used to know the appropriate deviation of the observable band positions from the scaled harmonic frequencies at a given level of theory. These considerations should always be taken into account whenever the experimental data is interpreted.

The dataset and scripts we used for quantum‐chemical calculations, data extraction, and scale factor fitting are available on Zenodo (https://doi.org/10.5281/zenodo.11174948). This data can be used to obtain the scale factors for other levels of theory.

## Appendix

We perform LSQ fitting of the parameter *s* by minimizing the following function[^72^, ^73^]

(6) $$\Phi \left(s\right)=\frac{1}{2}\sum_{i=1}^{N}\frac{{\left(s\cdot x_{i}-y_{i}\right)}^{2}}{\sigma_{0}^{2}},$$

where *N* is the total number of theoretical and experimental values, *x* is the scaled property, *y* is the reference data point, index 1≤i≤N enumerates the data points, and *σ* is the initially unknown uncertainty of all data points. In the case of absolute scale factor fitting, $x_{i}=\nu_{i}^{\left(\mathrm{calc}\right)}$ and $y_{i}=\nu_{i}^{\left(\exp\right)}$ , while for relative scale factor fitting, $x_{i}=\nu_{i}^{\left(\mathrm{calc}\right)}/\nu_{i}^{\left(\exp\right)}$ and $y_{i}=1$ .

The function $\Phi \left(s\right)$ can be interpreted through the maximal likelihood principle as the negative logarithm of the probability *p*(*s*) of the solution *s*, namely[^72^, ^73^]

(7) $$\Phi \left(s\right)=\ln\left(𝒩\right)-\ln\left(p\left(s\right)\right),$$

where 𝒩 is the normalization constant.

We can find the minimal value of the function from Equation 6 as $d\Phi \left(s\right)/ds=0$ , which gives the solution[^72^, ^73^]

(8) $$s_{\min}=\frac{S_{xy}}{S_{xx}},$$

where $S_{xy}=\sum_{i=1}^{N}x_{i}y_{i}$ and $S_{xx}=\sum_{i=1}^{N}x_{i}^{2}$ . Now, we can estimate the value of uncertainty *σ* through the minimal value of the RMSD given as

(9) $$\sigma_{0}^{2}={\mathrm{RMSD}}^{2}=\frac{1}{N}\sum_{i=1}^{N}{\left(s_{\min}\cdot x_{i}-y_{i}\right)}^{2}=\frac{1}{N}\left(S_{yy}-\frac{S_{xy}^{2}}{S_{xx}}\right),$$

where $S_{xy}=\sum_{i=1}^{N}y_{i}^{2}$ .

Representing *s* as $s=s_{\min}+\delta s$ , where *δs* is the deviation from the optimal LSQ solution (Equation 8), we can rewrite the original function $\Phi \left(s\right)$ (Equation 6) as[^72^, ^73^]

(10) $$\Phi \left(\delta s\right)=\frac{N}{2}+\frac{S_{xx}\delta s^{2}}{2\sigma_{0}^{2}}=\frac{N}{2}+\frac{\delta s^{2}}{2\sigma_{s}^{2}}.$$

Substitution of this equation into Equation 7 provides that the probability distribution of the deviation *δs* is a normal distribution with variance *σ_s_* given as

(11) $$\sigma_{s}^{2}=\frac{\sigma_{0}^{2}}{S_{xx}}.$$

Combining Equation 8 and 11 provides us with a final LSQ solution for scale factor *s* as $s=s_{0}\pm \sigma_{s}$ .

## Conflict of Interests

The authors declare no conflict of interest.

4

## Supporting information

As a service to our authors and readers, this journal provides supporting information supplied by the authors. Such materials are peer reviewed and may be re‐organized for online delivery, but are not copy‐edited or typeset. Technical support issues arising from supporting information (other than missing files) should be addressed to the authors.

Supporting Information

## Data Availability

The quantum‐chemical calculations, database, and scripts used for obtaining and analyzing the data are avaiable on Zenodo (https://doi.org/10.5281/zenodo.11174948). Additional data visualization and detailed representation of the results are provided in the ESI.
