# Supplementary material for: Harmonic Scale Factors of Fundamental Transitions for Dispersion‐corrected Quantum Chemical Methods
Source: Chemphyschem. 2024 Oct 28;25(23):e202400547. doi: 10.1002/cphc.202400547 (PMC11614367; doi:10.1002/cphc.202400547)
Supplement: Supplementary file 1 — Supporting Information [file CPHC-25-e202400547-s001.pdf]

ChemPhysChem

Supporting Information

Harmonic Scale Factors of Fundamental Transitions for Dispersion-corrected Quantum Chemical Methods

Denis S. Tikhonov,* Igor Gordiy, Danila A. Iakovlev, Alisa A. Gorislav, Mikhail A. Kalinin, Sergei A. Nikolenko, Ksenia M. Malaskeevich, Karina Yureva, Nikita A. Matsokin, and Melanie Schnell

Supporting Information:

Harmonic scale factors of fundamental transitions for dispersion-corrected quantum chemical methods

Contents

1 Scale factors statistics **S-2**

1.1 Numerical data S-2

1.2 Histograms S-6

1.2.1 Absolute deviations S-6

1.2.2 Relative deviations S-11

2 Illustrative examples **S-16**

2.1 Pyrene S-16

2.2 Protonated LeuEnk S-22

2.2.1 FELIX spectrum S-23

2.2.2 CLIO spectrum S-26

2.3 Doubly protonated conformer A of gramicidin S S-29

1 Scale factors statistics

1.1 Numerical data

Table S1 Scale factors obtained for the 27 dispersion-corrected quantum-chemical methods.

Method	Absolute scale factor s_{abs}	Relative scale factor s_{rel}
BLYP-D3BJ/6-31G	0.99110 ± 0.00063	1.01754 ± 0.00160
BLYP-D3BJ/def2-SVP	0.99716 ± 0.00035	1.01325 ± 0.00113
BLYP-D3BJ/def2-TZVP	0.99598 ± 0.00035	1.01683 ± 0.00105
BLYP-D4/6-31G	0.99123 ± 0.00063	1.01739 ± 0.00160
BLYP-D4/def2-SVP	0.99727 ± 0.00035	1.01312 ± 0.00114
BLYP-D4/def2-TZVP	0.99611 ± 0.00035	1.01679 ± 0.00105
PBE-D3BJ/6-31G	0.98478 ± 0.00059	1.01017 ± 0.00152
PBE-D3BJ/def2-SVP	0.99035 ± 0.00036	1.00564 ± 0.00109
PBE-D3BJ/def2-TZVP	0.99234 ± 0.00033	1.01057 ± 0.00100
PBE-D4/6-31G	0.98491 ± 0.00058	1.00960 ± 0.00155
PBE-D4/def2-SVP	0.99046 ± 0.00036	1.00522 ± 0.00112
PBE-D4/def2-TZVP	0.99246 ± 0.00033	1.01023 ± 0.00103
B3LYP-D3BJ/6-31G	0.96110 ± 0.00054	0.98111 ± 0.00143
B3LYP-D3BJ/def2-SVP	0.96637 ± 0.00030	0.97548 ± 0.00103
B3LYP-D3BJ/def2-TZVP	0.96712 ± 0.00026	0.97854 ± 0.00093
B3LYP-D4/6-31G	0.96132 ± 0.00054	0.98143 ± 0.00142
B3LYP-D4/def2-SVP	0.96657 ± 0.00030	0.97586 ± 0.00102
B3LYP-D4/def2-TZVP	0.96733 ± 0.00026	0.97897 ± 0.00093
PBE0-D3BJ/6-31G	0.95092 ± 0.00049	0.96935 ± 0.00132
PBE0-D3BJ/def2-SVP	0.95550 ± 0.00032	0.96308 ± 0.00099
PBE0-D3BJ/def2-TZVP	0.95908 ± 0.00027	0.96723 ± 0.00092
PBE0-D4/6-31G	0.95100 ± 0.00049	0.96931 ± 0.00133
PBE0-D4/def2-SVP	0.95557 ± 0.00032	0.96303 ± 0.00100
PBE0-D4/def2-TZVP	0.95915 ± 0.00027	0.96720 ± 0.00093
HF-3c	0.83998 ± 0.00060	0.85826 ± 0.00140
r^{2}SCAN-3c	0.93293 ± 0.00026	0.93468 ± 0.00094
r^{2}SCAN-3c	0.96878 ± 0.00029	0.98155 ± 0.00106

Table S2 Absolute root-mean-square deviations (aRMSD) for unscaled/s_{abs}-scaled/s_{rel}-scaled datasets. All values are in cm^{-1}.

Method	Unscaled	s_{abs}-scaled	s_{rel}-scaled
BLYP-D3BJ/6-31G	74	72	84
BLYP-D3BJ/def2-SVP	41	41	48
BLYP-D3BJ/def2-TZVP	42	41	52
BLYP-D4/6-31G	74	72	83
BLYP-D4/def2-SVP	41	41	48
BLYP-D4/def2-TZVP	41	41	52
PBE-D3BJ/6-31G	72	67	79
PBE-D3BJ/def2-SVP	45	42	48
PBE-D3BJ/def2-TZVP	40	39	48
PBE-D4/6-31G	72	67	78
PBE-D4/def2-SVP	45	42	48
PBE-D4/def2-TZVP	40	39	47
B3LYP-D3BJ/6-31G	90	63	71
B3LYP-D3BJ/def2-SVP	65	36	39
B3LYP-D3BJ/def2-TZVP	62	32	37
B3LYP-D4/6-31G	90	63	72
B3LYP-D4/def2-SVP	64	36	39
B3LYP-D4/def2-TZVP	61	32	37
PBE0-D3BJ/6-31G	101	59	66
PBE0-D3BJ/def2-SVP	82	39	41
PBE0-D3BJ/def2-TZVP	74	33	36
PBE0-D4/6-31G	101	59	66
PBE0-D4/def2-SVP	82	39	41
PBE0-D4/def2-TZVP	74	33	36
HF-3c	305	82	89
r^{2}SCAN-3c	116	33	33
r^{2}SCAN-3c	61	35	41

Table S3 Relative root-mean-square deviations (rRMSD) for unscaled/s_{abs}-scaled/s_{rel}-scaled datasets. Values are dimensionless.

Method	Unscaled	s_{abs}-scaled	s_{rel}-scaled
BLYP-D3BJ/6-31G	0.11282	0.11446	0.11151
BLYP-D3BJ/def2-SVP	0.08529	0.08576	0.08429
BLYP-D3BJ/def2-TZVP	0.07993	0.08084	0.07821
BLYP-D4/6-31G	0.11328	0.11487	0.11200
BLYP-D4/def2-SVP	0.08614	0.08658	0.08516
BLYP-D4/def2-TZVP	0.08030	0.08117	0.07859
PBE-D3BJ/6-31G	0.10712	0.10954	0.10665
PBE-D3BJ/def2-SVP	0.08176	0.08296	0.08157
PBE-D3BJ/def2-TZVP	0.07592	0.07732	0.07520
PBE-D4/6-31G	0.10927	0.11154	0.10886
PBE-D4/def2-SVP	0.08423	0.08533	0.08407
PBE-D4/def2-TZVP	0.07803	0.07934	0.07738
B3LYP-D3BJ/6-31G	0.10514	0.10535	0.10338
B3LYP-D3BJ/def2-SVP	0.08360	0.08030	0.07975
B3LYP-D3BJ/def2-TZVP	0.07555	0.07325	0.07232
B3LYP-D4/6-31G	0.10491	0.10520	0.10321
B3LYP-D4/def2-SVP	0.08327	0.08010	0.07954
B3LYP-D4/def2-TZVP	0.07489	0.07273	0.07176
PBE0-D3BJ/6-31G	0.10206	0.09892	0.09709
PBE0-D3BJ/def2-SVP	0.08650	0.07799	0.07759
PBE0-D3BJ/def2-TZVP	0.07993	0.07292	0.07243
PBE0-D4/6-31G	0.10260	0.09943	0.09764
PBE0-D4/def2-SVP	0.08714	0.07867	0.07829
PBE0-D4/def2-TZVP	0.08043	0.07345	0.07298
HF-3c	0.20440	0.12393	0.12211
r^2SCAN-3c	0.10345	0.07648	0.07646
r^2SCAN-3c	0.08397	0.08287	0.08185

Table S4 Number of molecules (N_{mol}) and number of harmonic frequencies (N_{freq}) in dataset that was used to obtain scale factors for a given quantum-chemical method.

Method	N_{mol}	N_{freq}
BLYP-D3BJ/6-31G	341	5107
BLYP-D3BJ/def2-SVP	441	5778
BLYP-D3BJ/def2-TZVP	441	5778
BLYP-D4/6-31G	341	5107
BLYP-D4/def2-SVP	441	5778
BLYP-D4/def2-TZVP	441	5778
PBE-D3BJ/6-31G	341	5107
PBE-D3BJ/def2-SVP	440	5739
PBE-D3BJ/def2-TZVP	441	5778
PBE-D4/6-31G	341	5107
PBE-D4/def2-SVP	440	5739
PBE-D4/def2-TZVP	441	5778
B3LYP-D3BJ/6-31G	342	5113
B3LYP-D3BJ/def2-SVP	441	5778
B3LYP-D3BJ/def2-TZVP	441	5778
B3LYP-D4/6-31G	342	5113
B3LYP-D4/def2-SVP	441	5778
B3LYP-D4/def2-TZVP	441	5778
PBE0-D3BJ/6-31G	342	5113
PBE0-D3BJ/def2-SVP	439	5706
PBE0-D3BJ/def2-TZVP	441	5778
PBE0-D4/6-31G	342	5113
PBE0-D4/def2-SVP	439	5706
PBE0-D4/def2-TZVP	441	5778
HF-3c	436	5667
r^2SCAN-3c	440	5769
r^2SCAN-3c	440	5754

1.2 Histograms

1.2.1 Absolute deviations

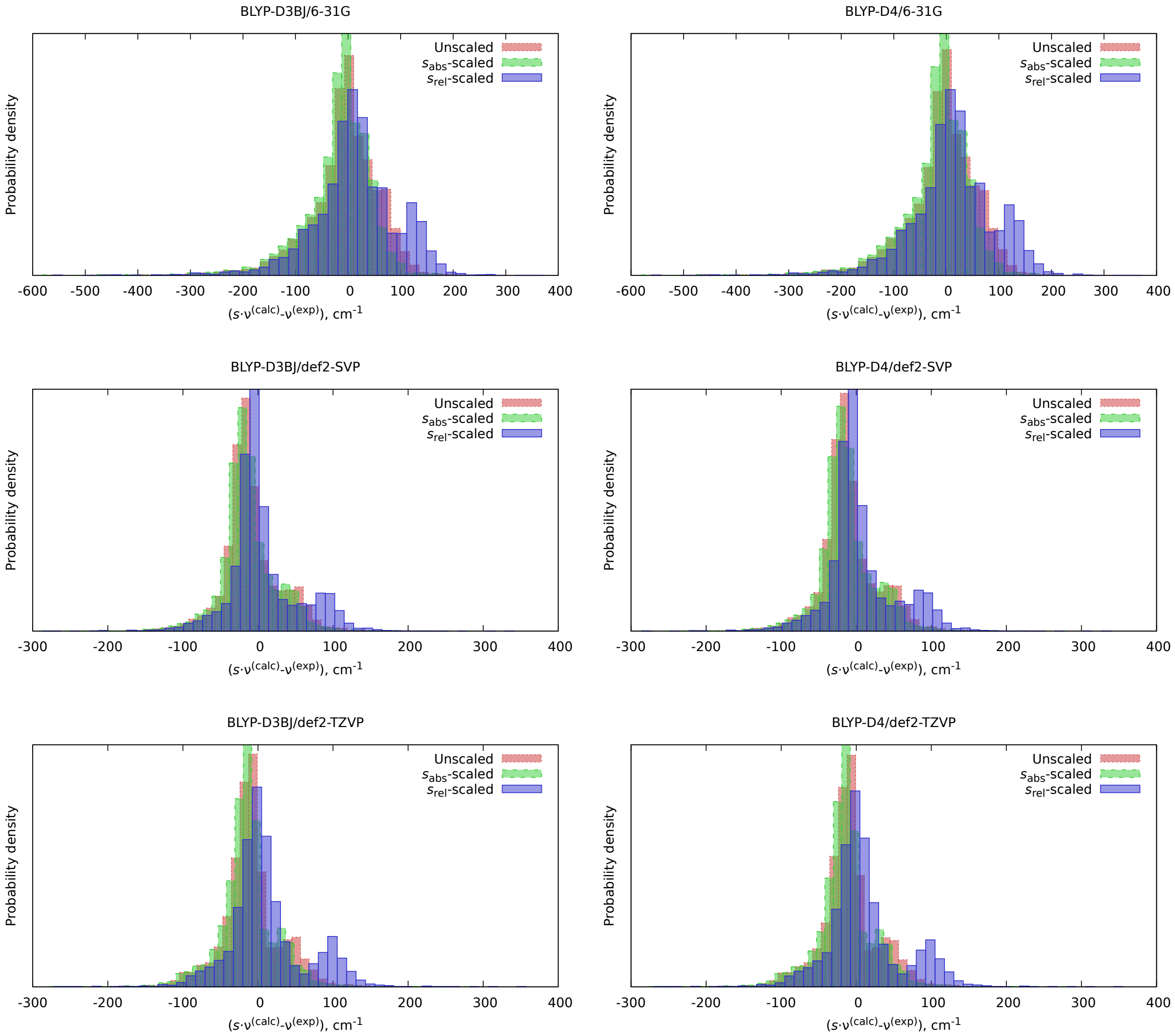

Figure S1 Absolute deviations of the unscaled/s_{abs}-scaled/s_{rel}-scaled frequencies from the experimental fundamental transitions in the dataset for BLYP-based methods.

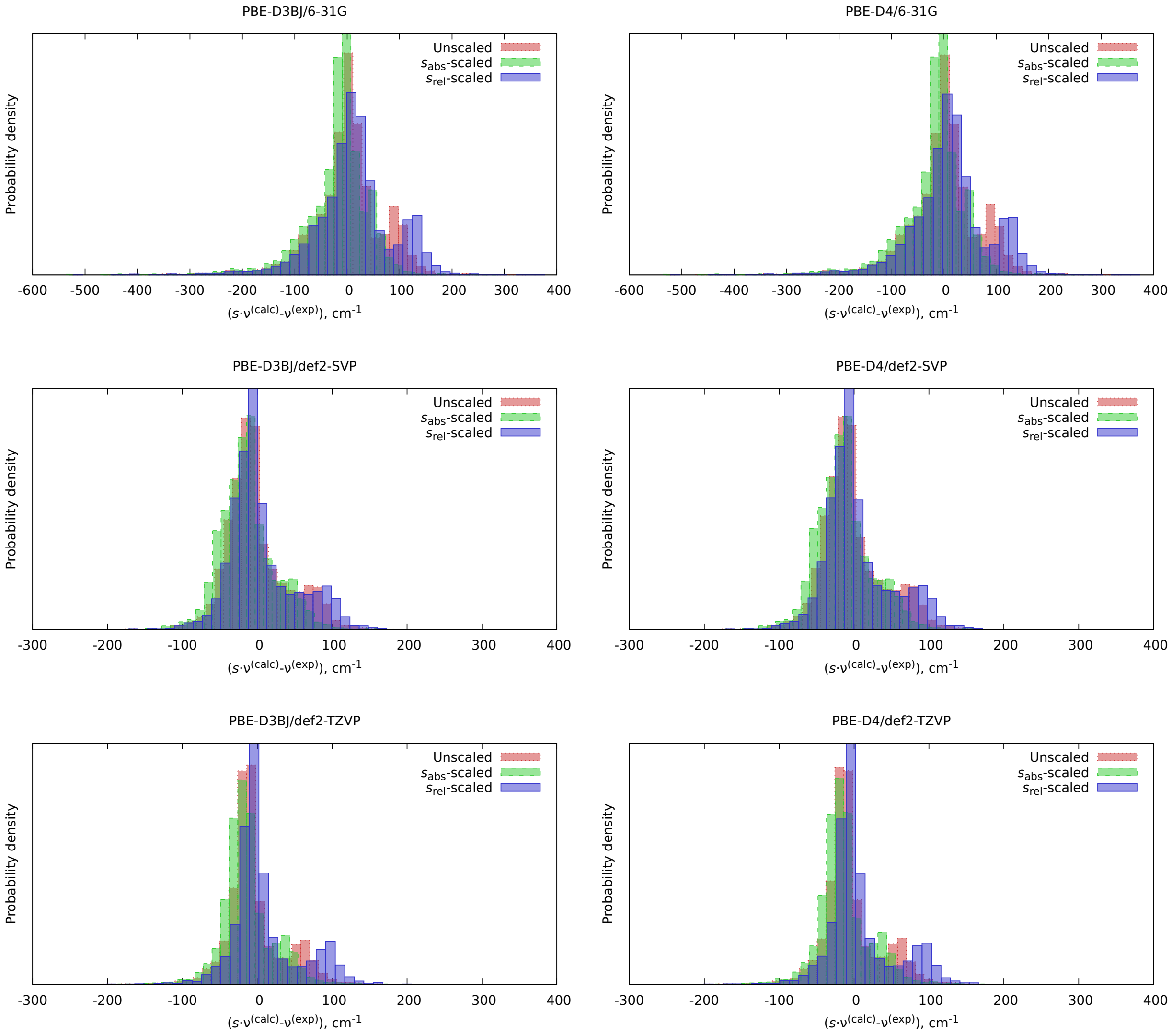

Figure S2 Absolute deviations of the unscaled/s_{abs}-scaled/s_{rel}-scaled frequencies from the experimental fundamental transitions in the dataset for PBE-based methods.

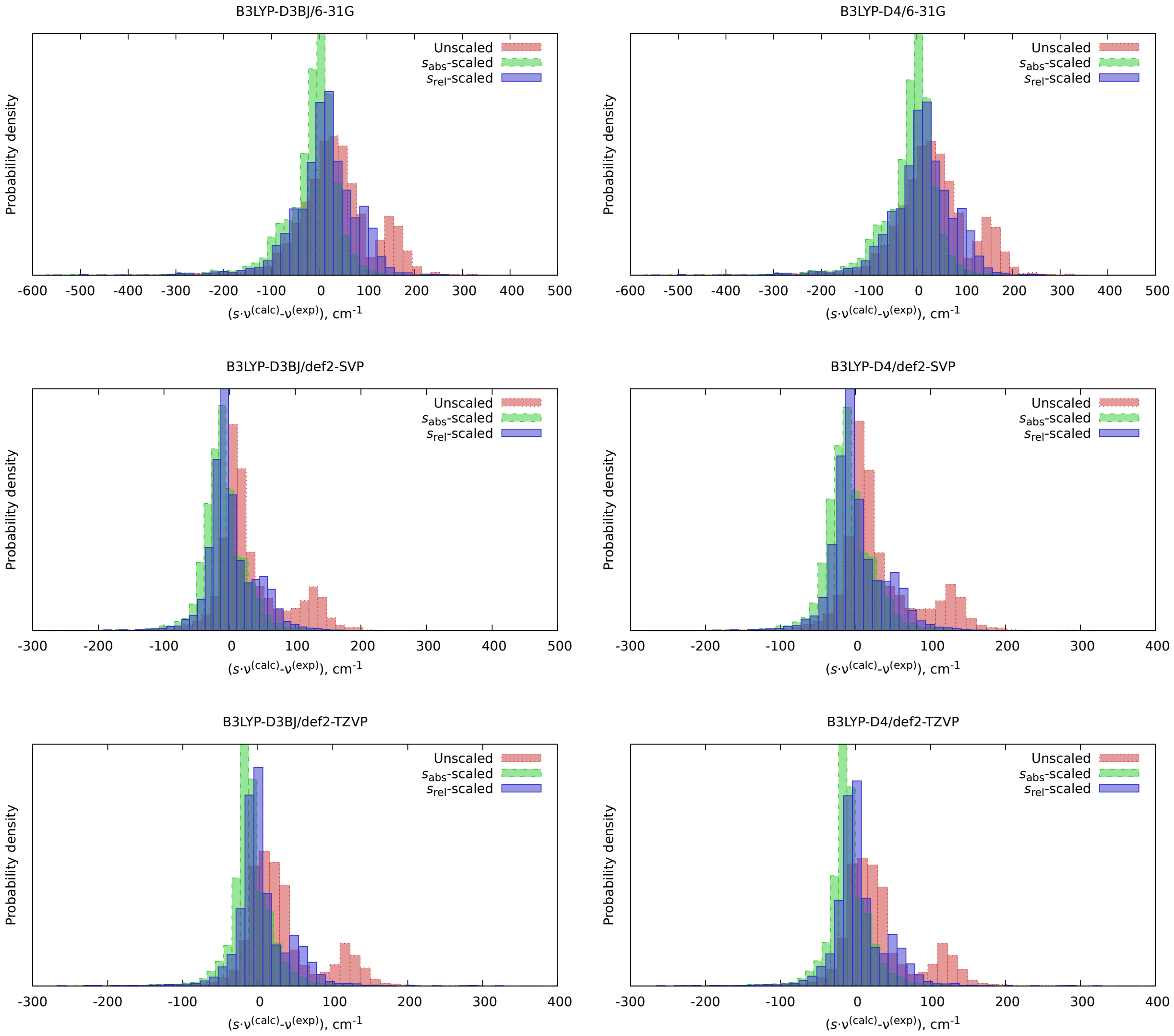

Figure S3 Absolute deviations of the unscaled/s_{abs}-scaled/s_{rel}-scaled frequencies from the experimental fundamental transitions in the dataset for B3LYP-based methods.

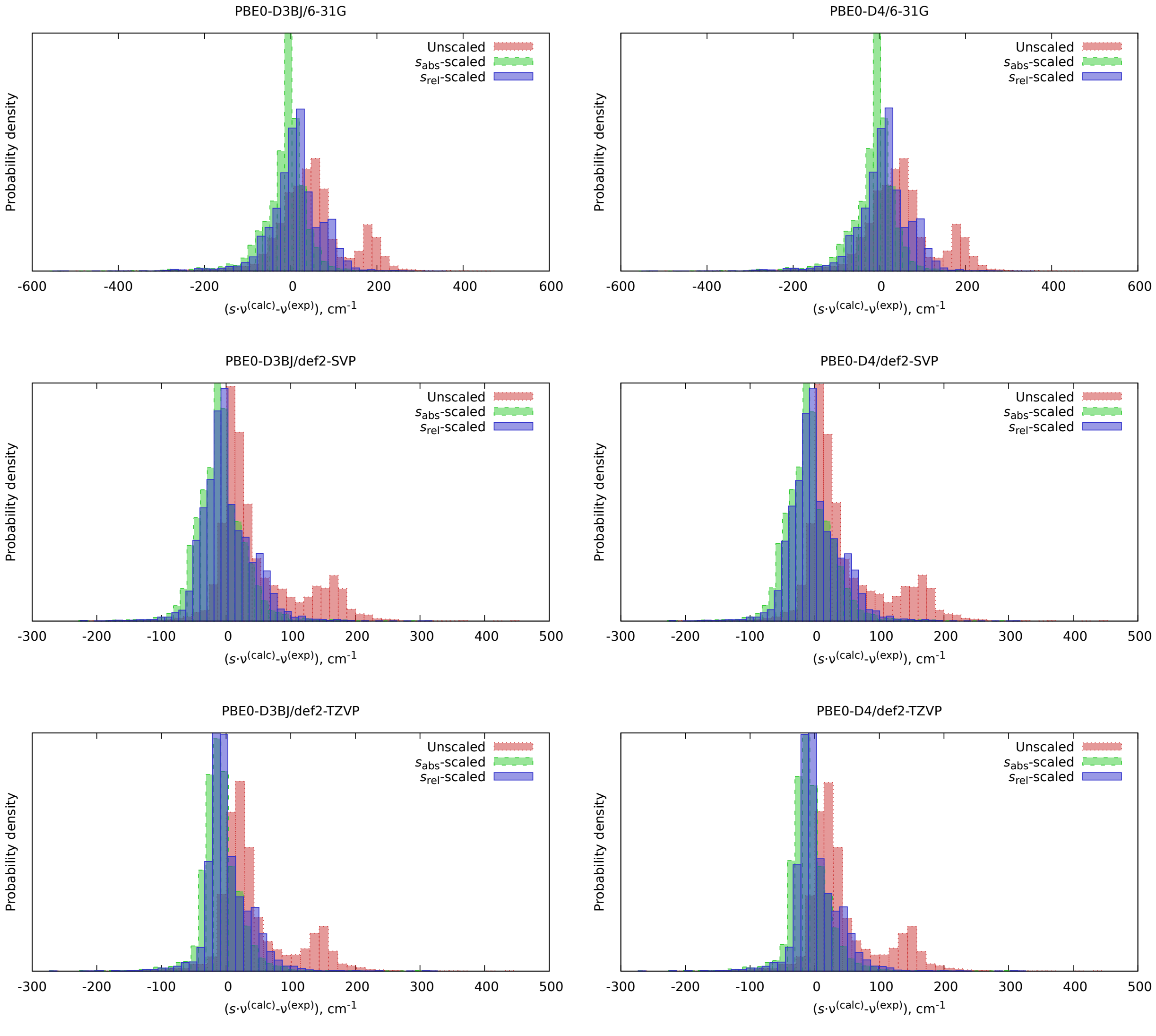

Figure S4 Absolute deviations of the unscaled/s_{abs}-scaled/s_{rel}-scaled frequencies from the experimental fundamental transitions in the dataset for PBE0-based methods.

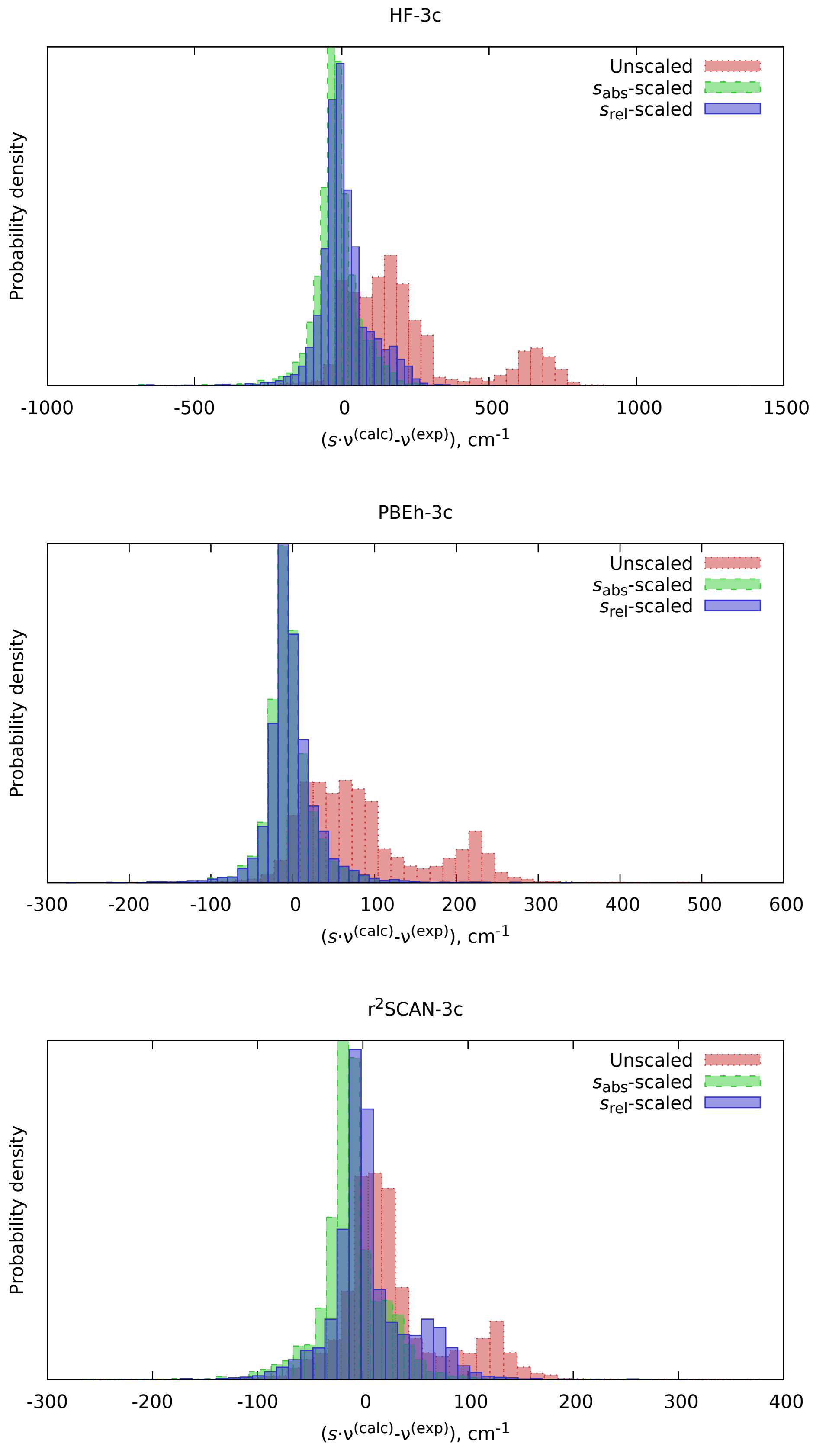

Figure S5 Absolute deviations of the unscaled/s_{abs}-scaled/s_{rel}-scaled frequencies from the experimental fundamental transitions in the dataset for 3c composite methods.

1.2.2 Relative deviations

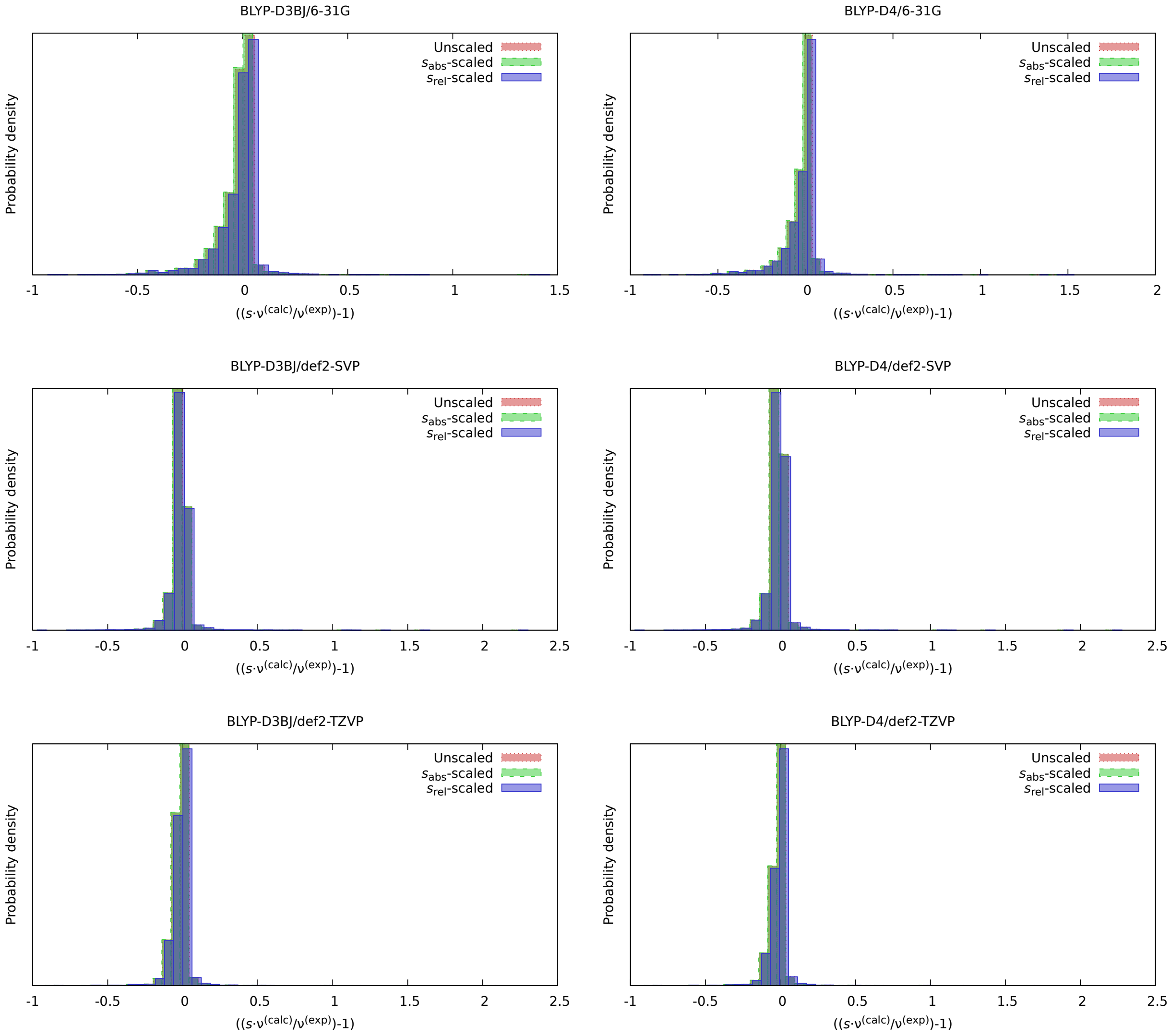

Figure S6 Relative deviations of the unscaled/s_{abs}-scaled/s_{rel}-scaled frequencies from the experimental fundamental transitions in the dataset for BLYP-based methods.

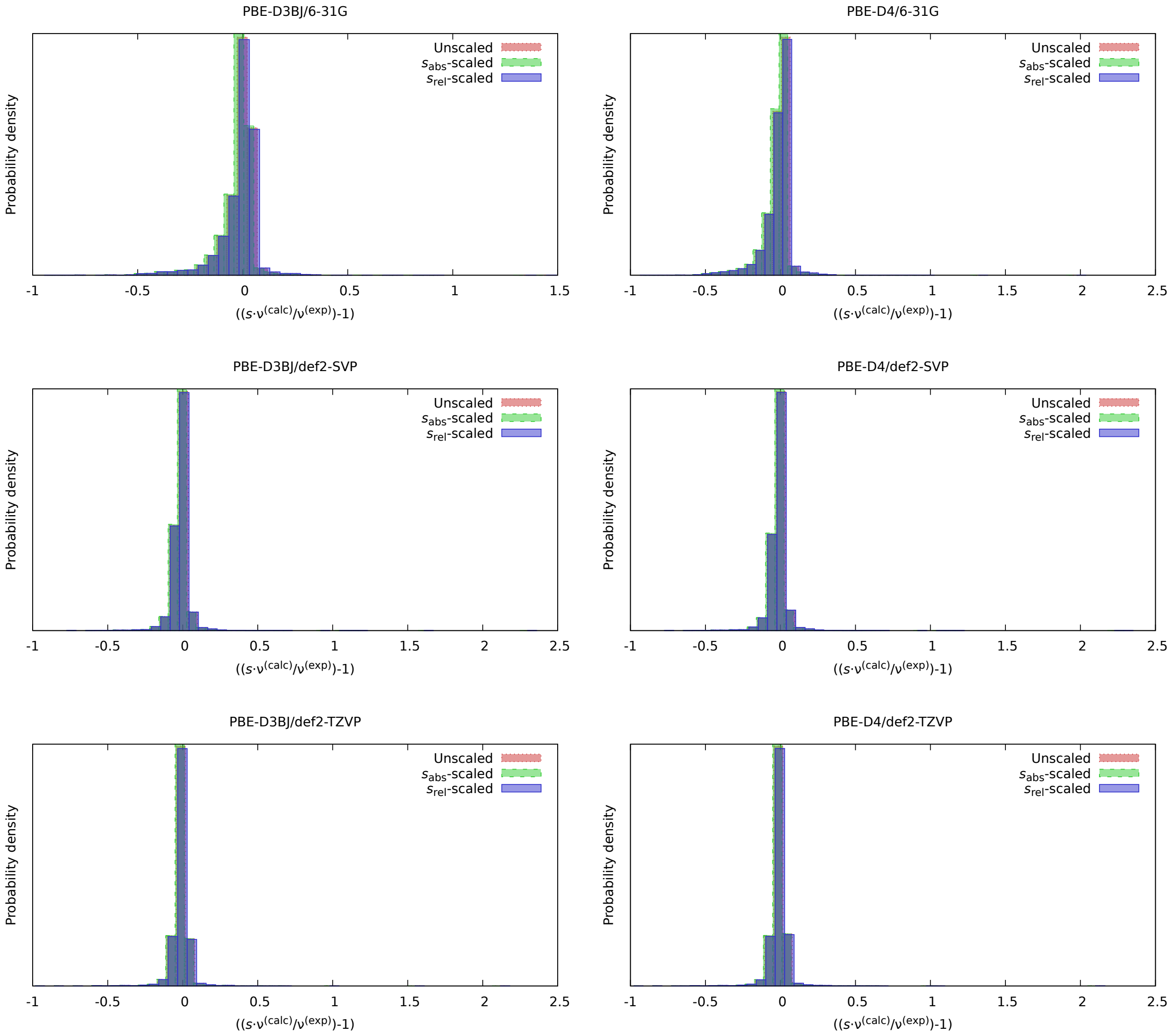

Figure S7 Relative deviations of the unscaled/s_{abs}-scaled/s_{rel}-scaled frequencies from the experimental fundamental transitions in the dataset for PBE-based methods.

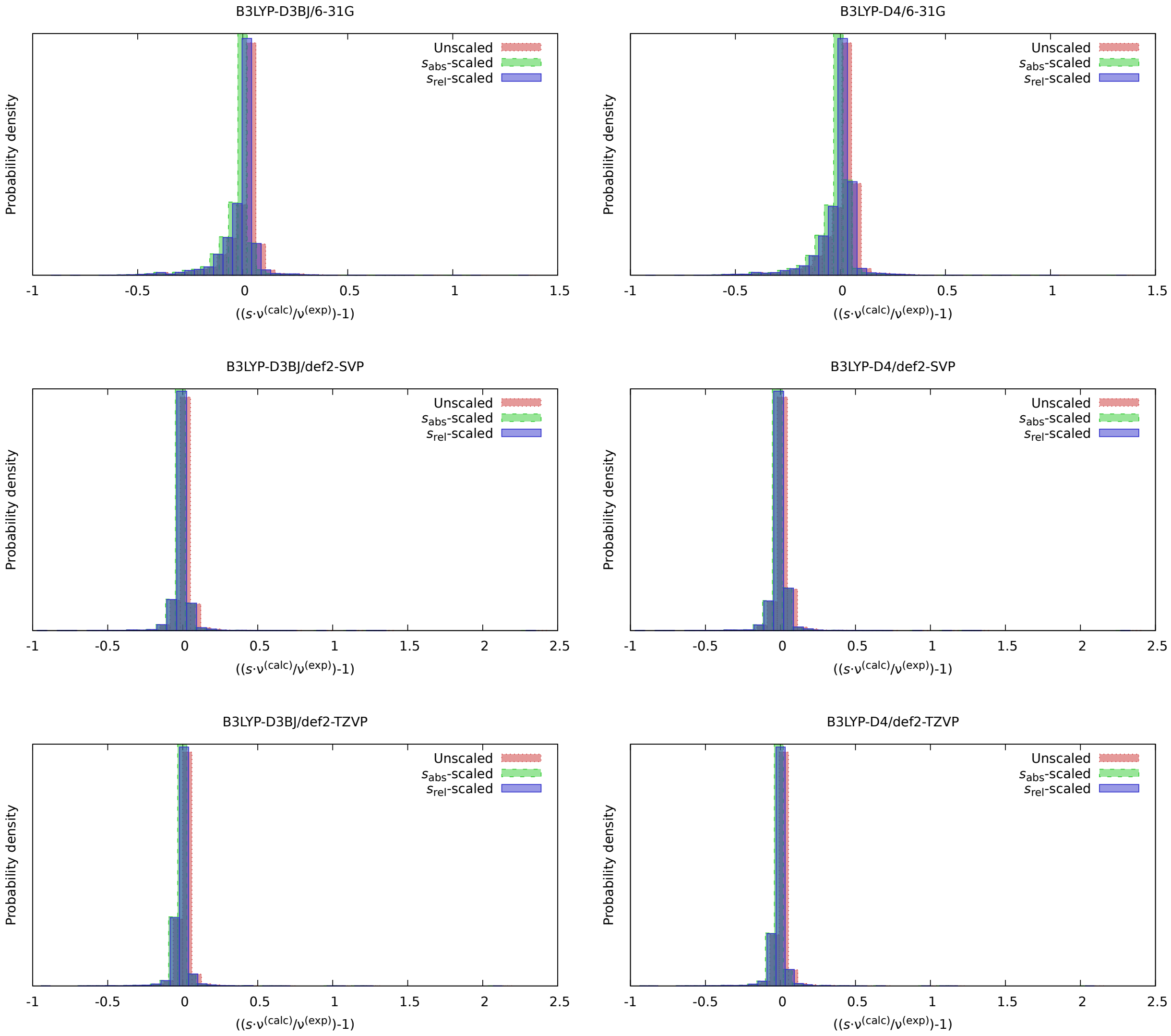

Figure S8 Relative deviations of the unscaled/s_{abs}-scaled/s_{rel}-scaled frequencies from the experimental fundamental transitions in the dataset for B3LYP-based methods.

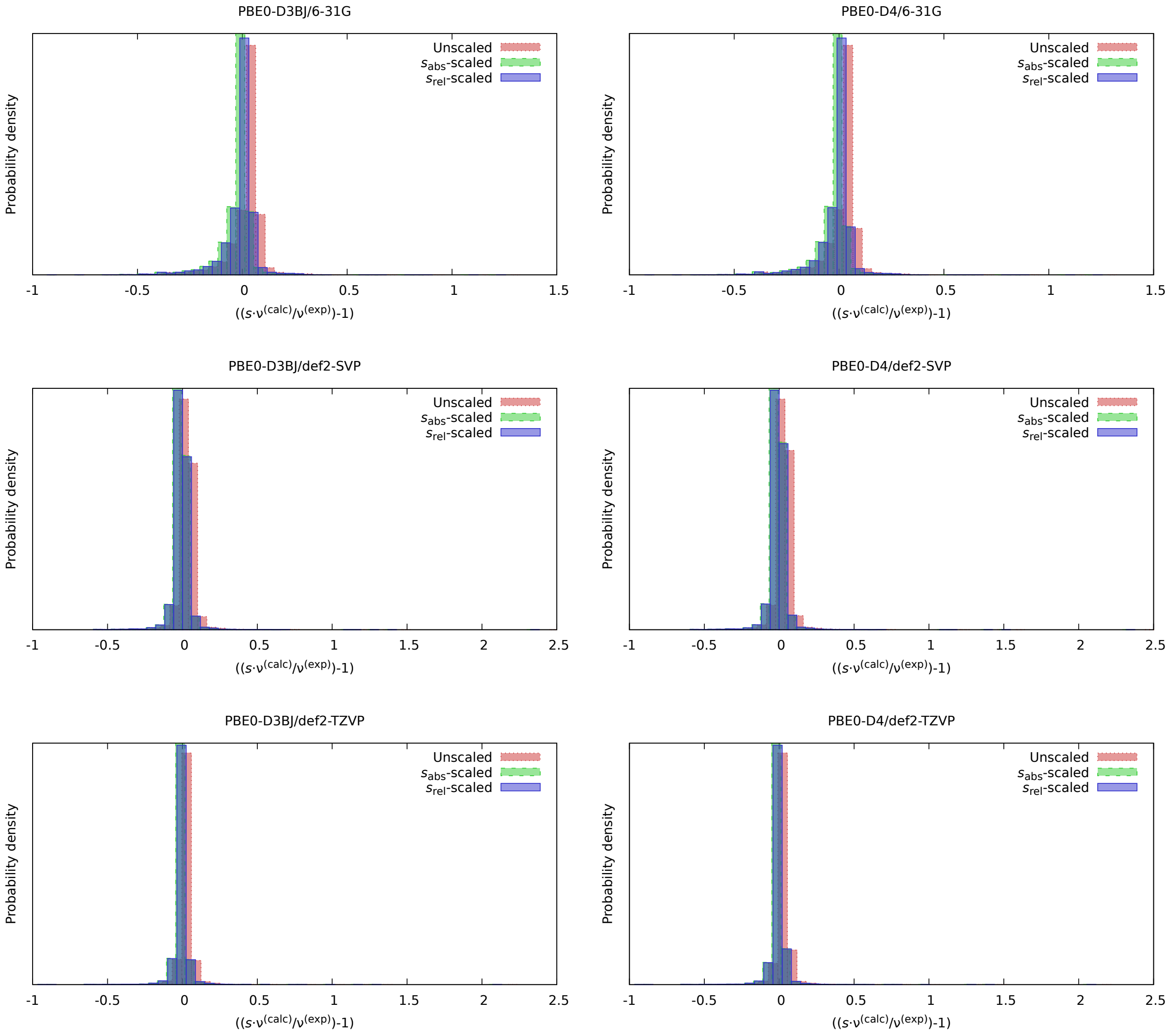

Figure S9 Relative deviations of the unscaled/s_{abs}-scaled/s_{rel}-scaled frequencies from the experimental fundamental transitions in the dataset for PBE0-based methods.

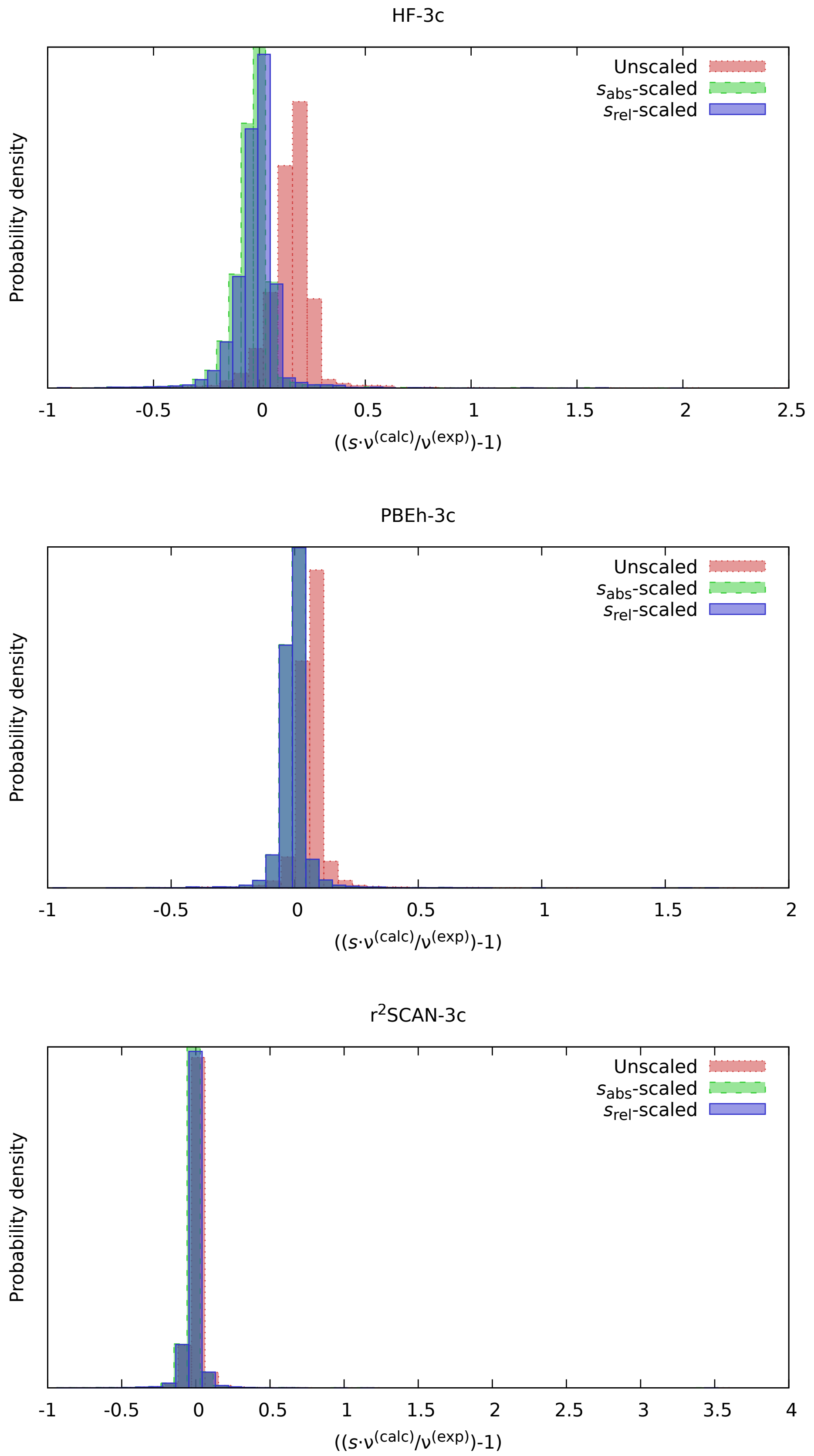

Figure S10 Relative deviations of the unscaled/s_{abs}-scaled/s_{rel}-scaled frequencies from the experimental fundamental transitions in the dataset for 3c composite methods.

2 Illustrative examples

2.1 Pyrene

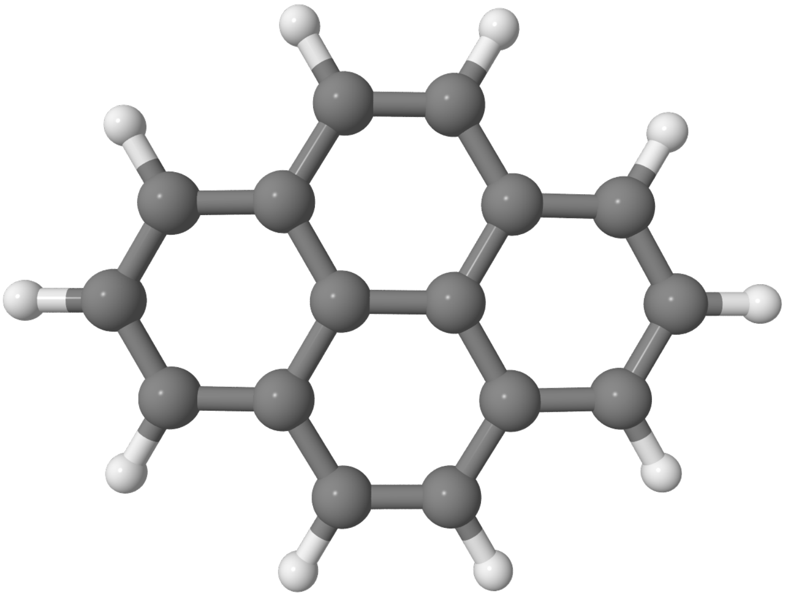

Figure S11 Molecular structure of pyrene.

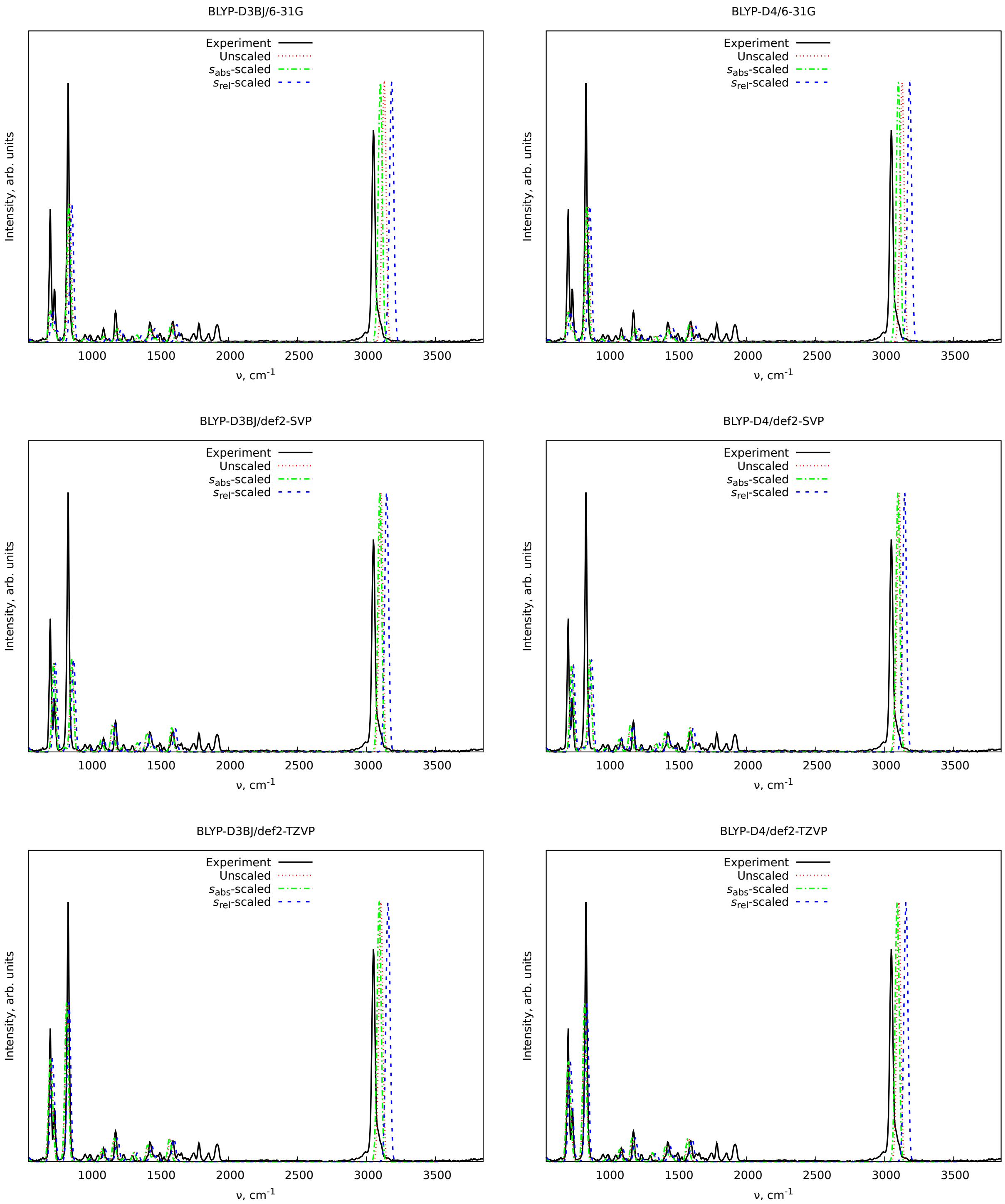

Figure S12 Unscaled and scaled harmonic and experimental spectra of pyrene. Theoretical harmonic spectra were obtained with BLYP-based methods.

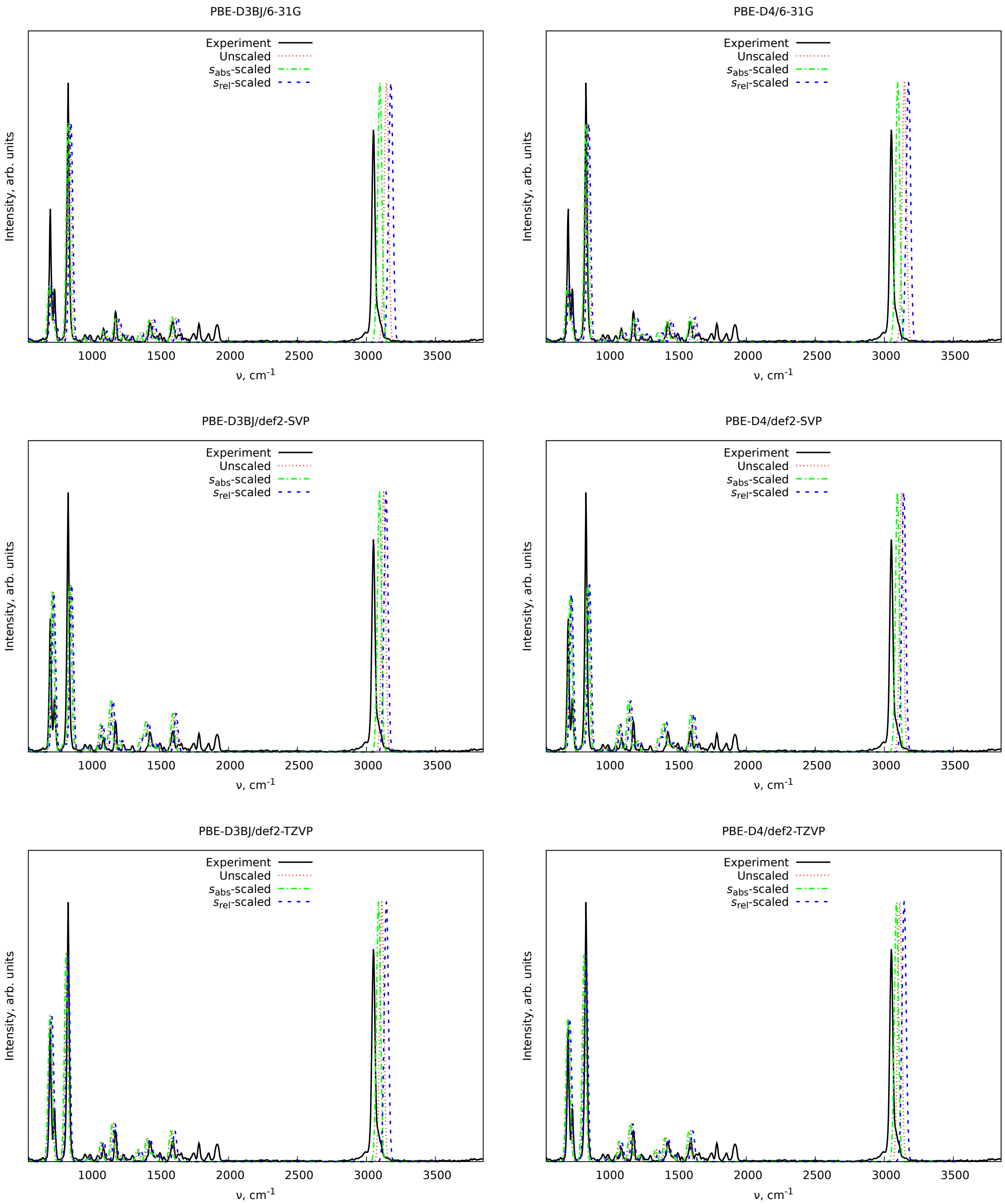

Figure S13 Unscaled and scaled harmonic and experimental spectra of pyrene. Theoretical harmonic spectra were obtained with PBE-based methods.

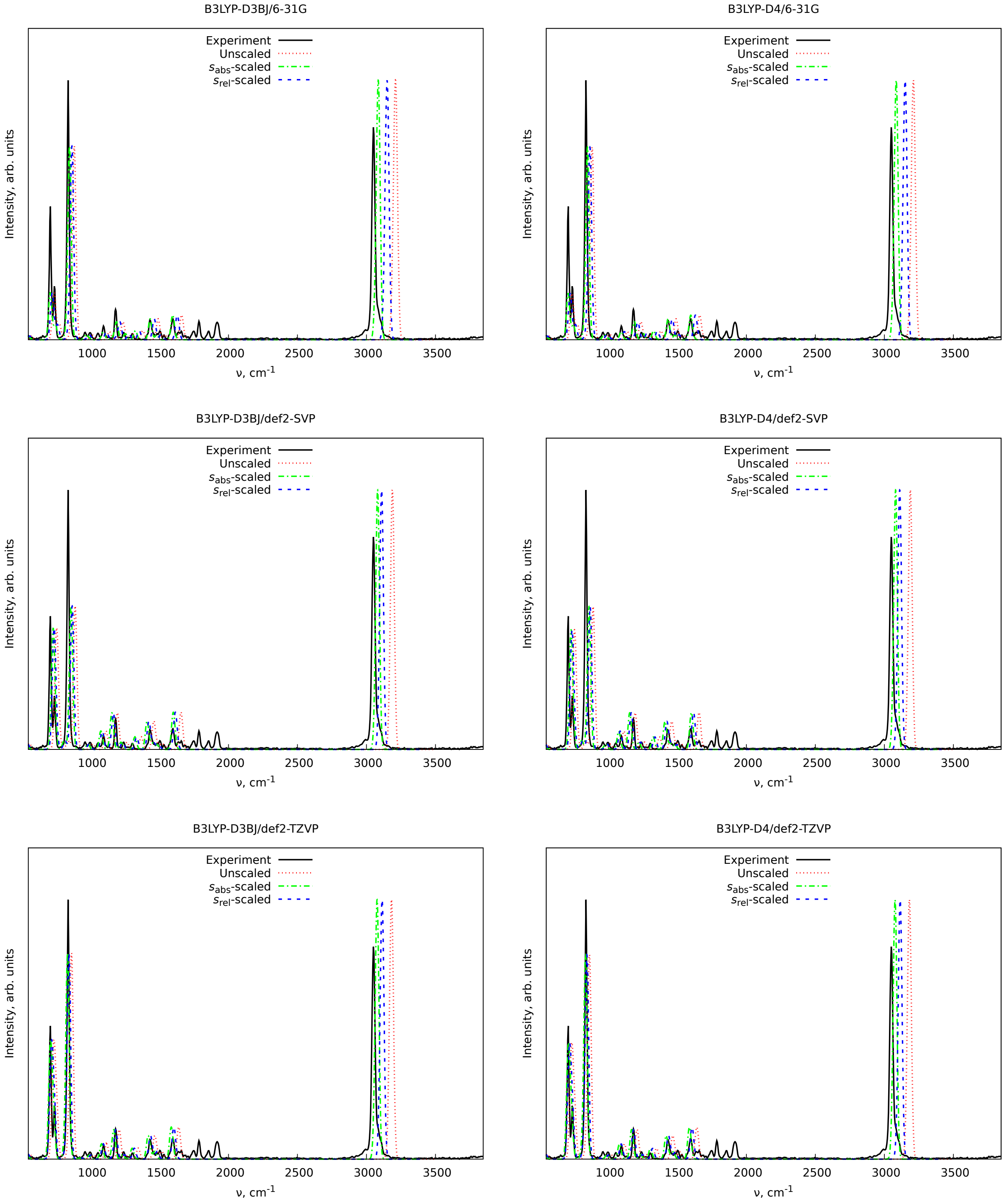

Figure S14 Unscaled and scaled harmonic and experimental spectra of pyrene. Theoretical harmonic spectra were obtained with B3LYP-based methods.

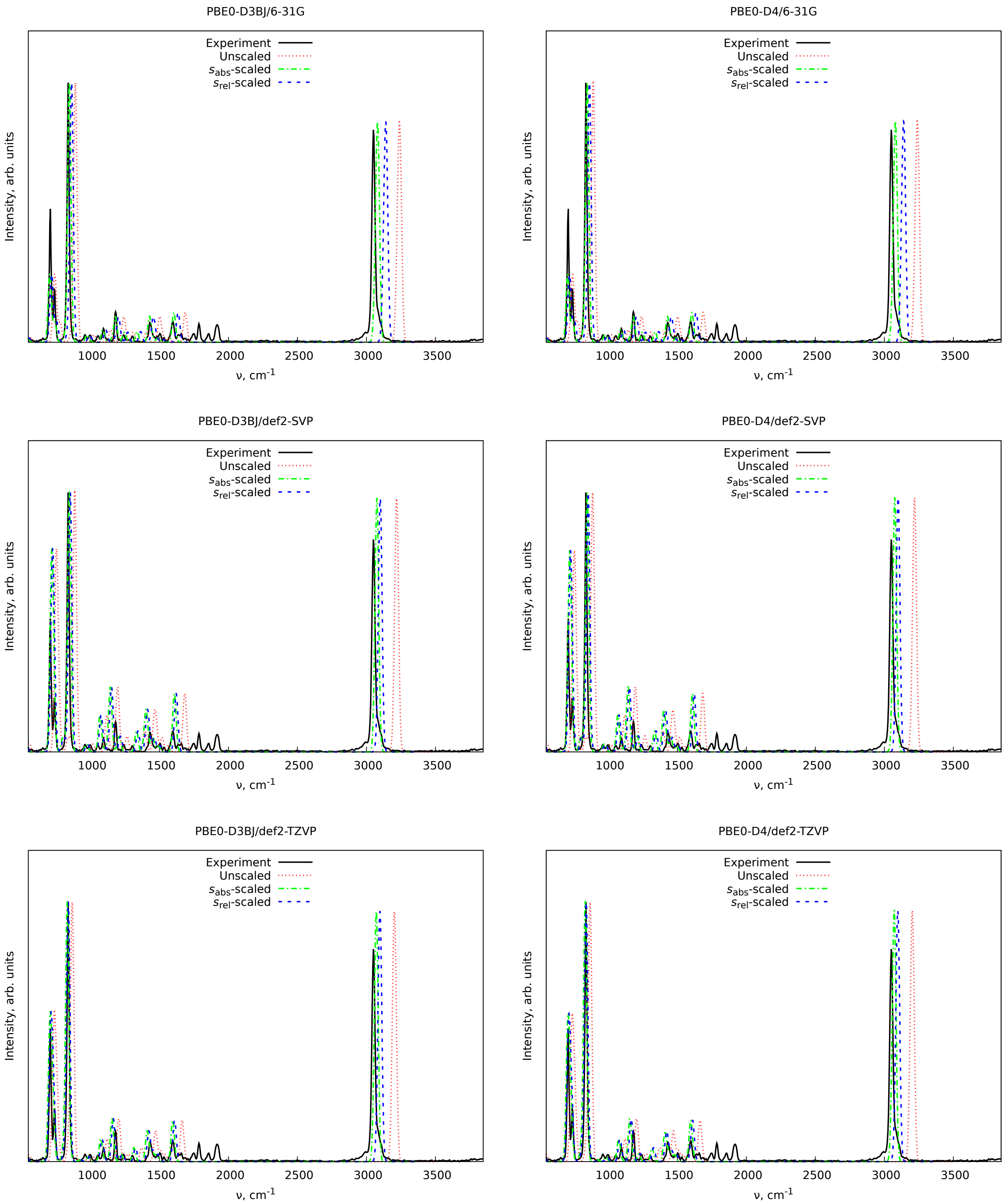

Figure S15 Unscaled and scaled harmonic and experimental spectra of pyrene. Theoretical harmonic spectra were obtained with PBE0-based methods.

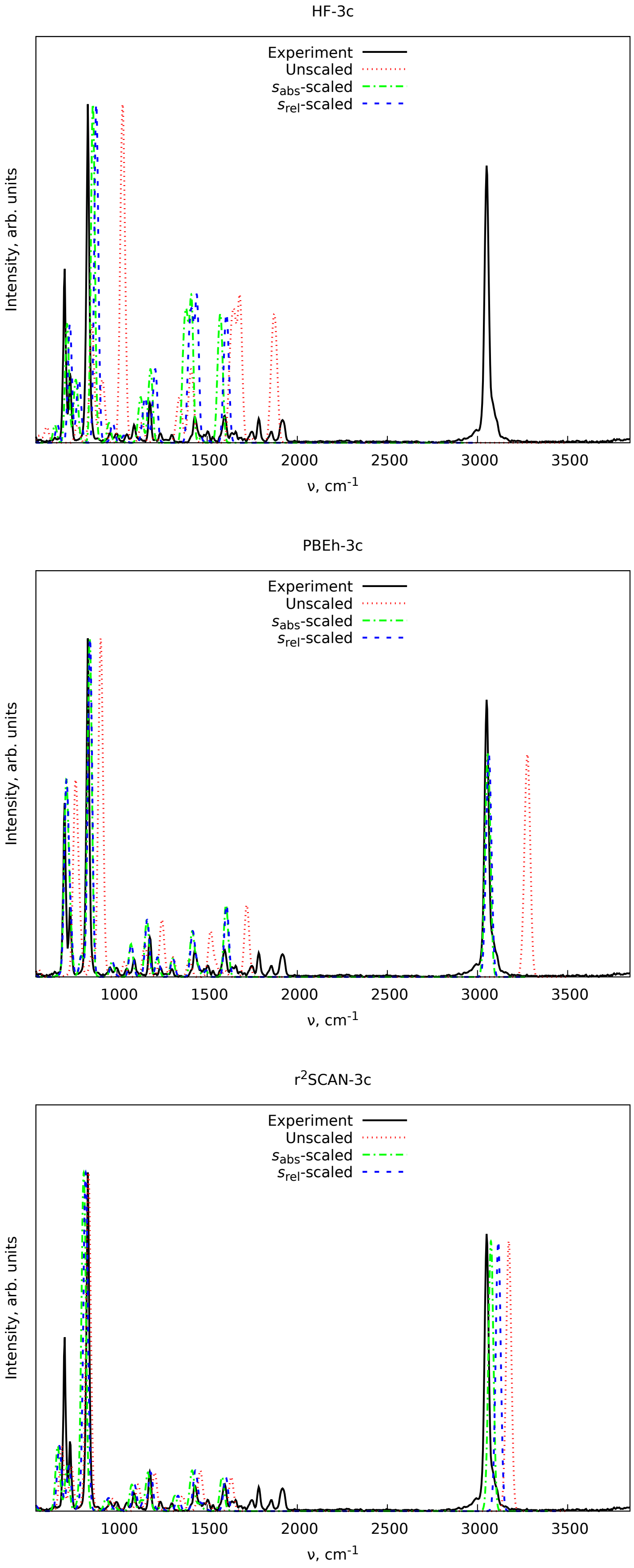

Figure S16 Unscaled and scaled harmonic and experimental spectra of pyrene. Theoretical harmonic spectra were obtained with 3c composite methods.

2.2 Protonated LeuEnk

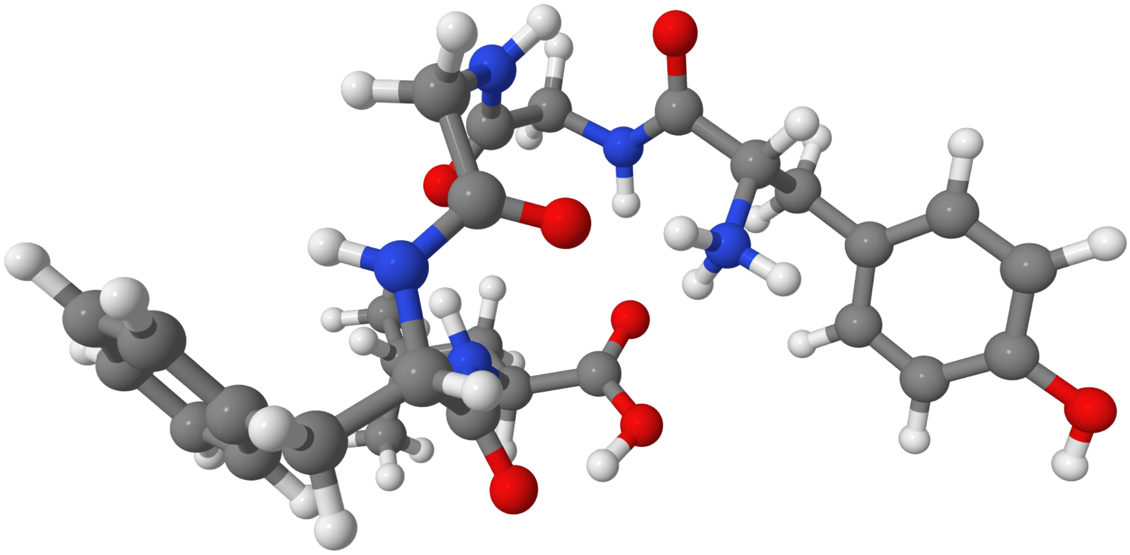

Figure S17 Molecular structure of the most stable protonated LeuEnk conformer.

2.2.1 FELIX spectrum

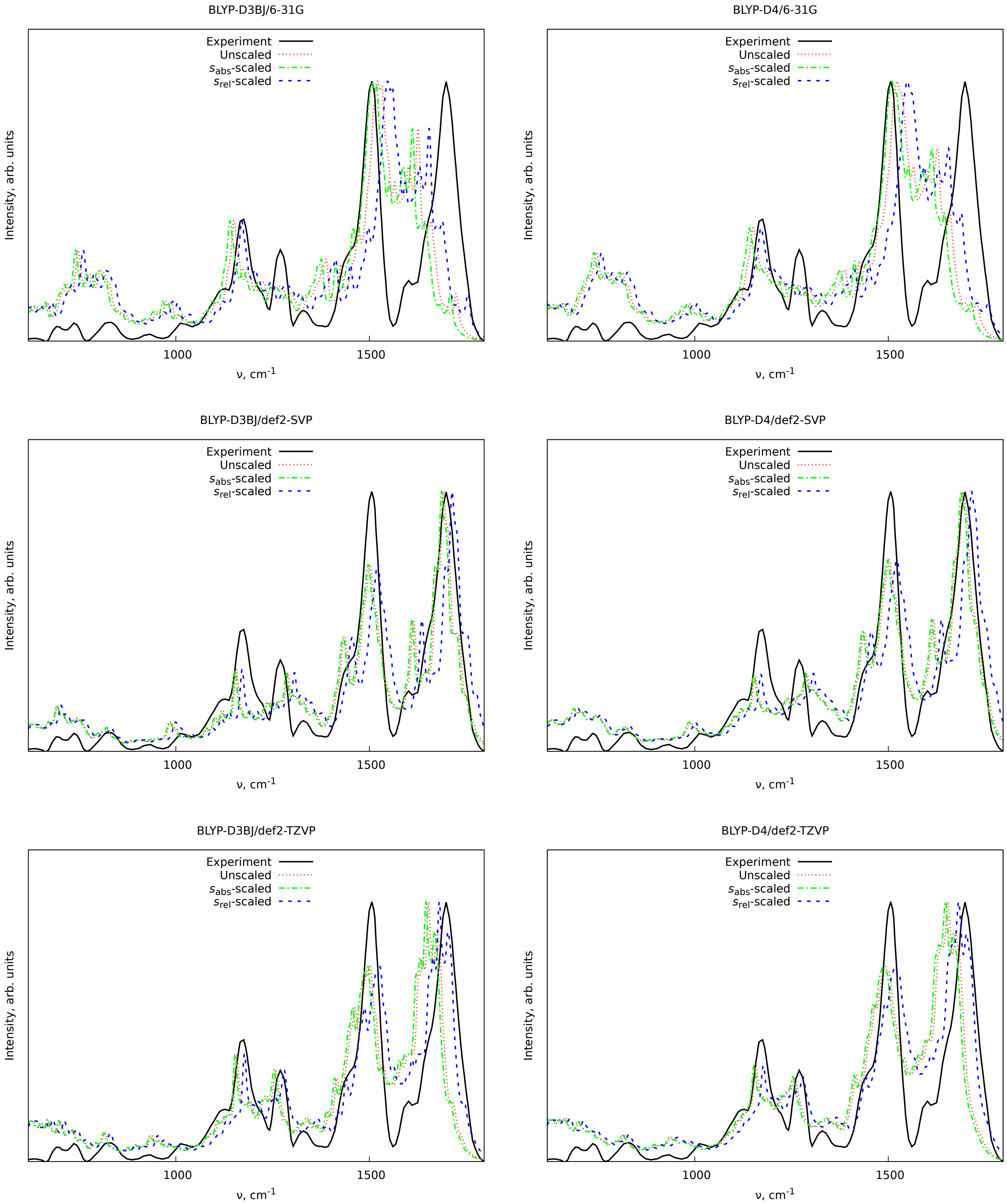

Figure S18 Unscaled and scaled harmonic and experimental (FELIX) spectra of leuenk. Theoretical harmonic spectra were obtained with BLYP-based methods.

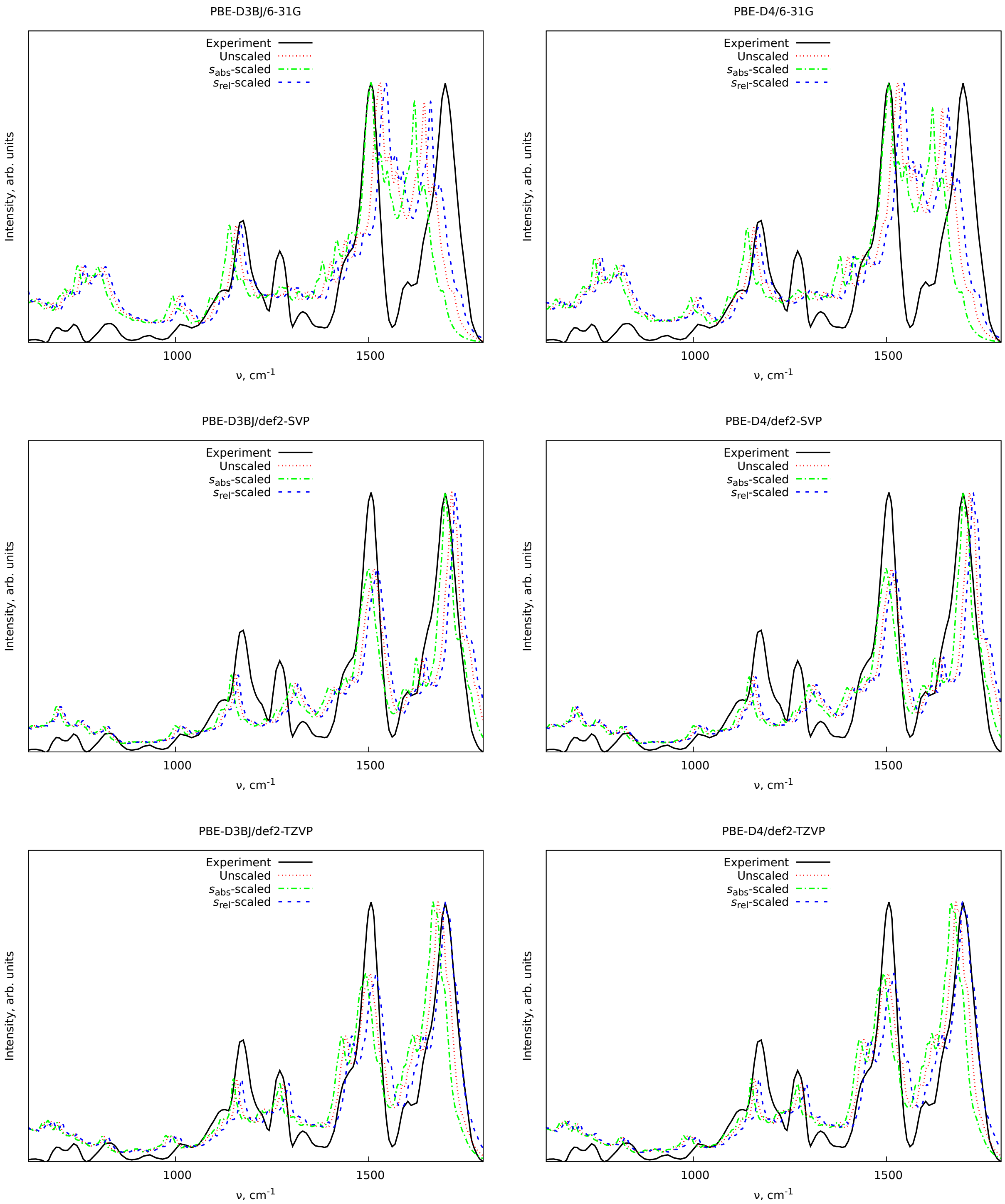

Figure S19 Unscaled and scaled harmonic and experimental (FELIX) spectra of leuenk. Theoretical harmonic spectra were obtained with PBE-based methods.

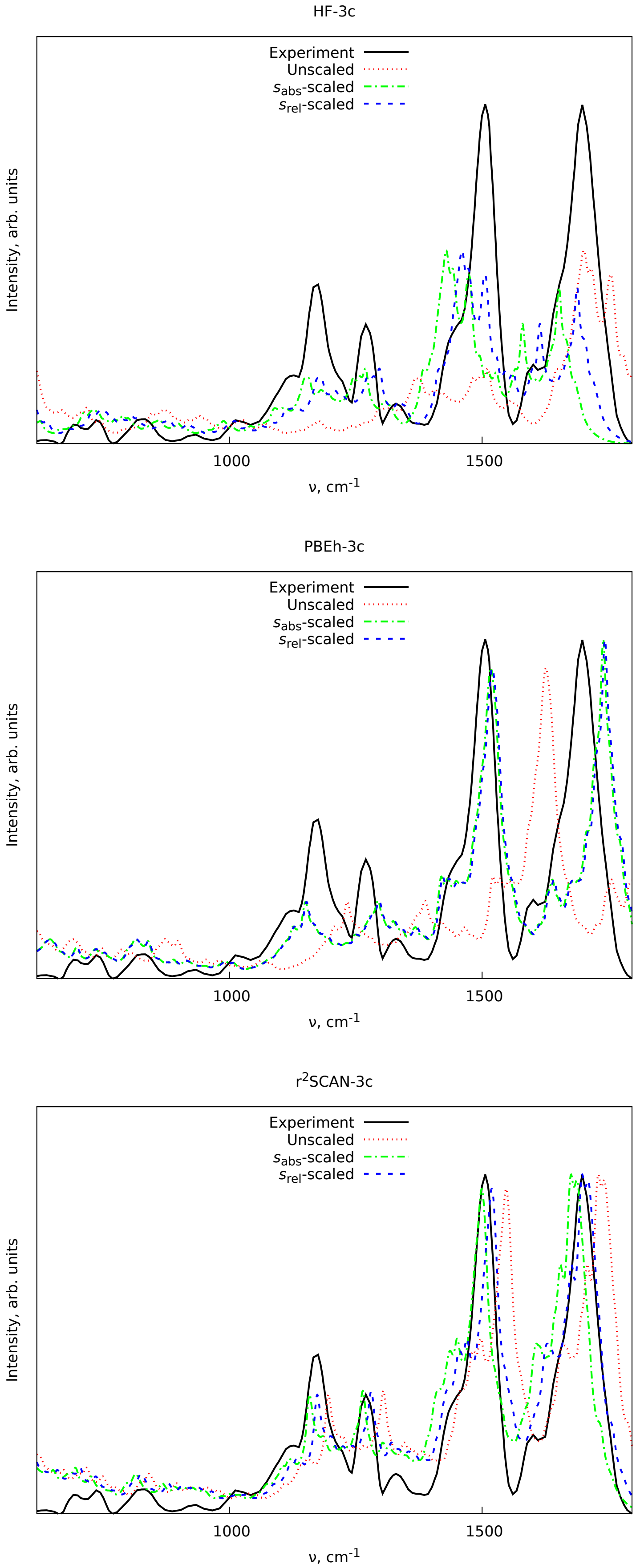

Figure S20 Unscaled and scaled harmonic and experimental (FELIX) spectra of leuenk. Theoretical harmonic spectra were obtained with 3c composite methods.

2.2.2 CLIO spectrum

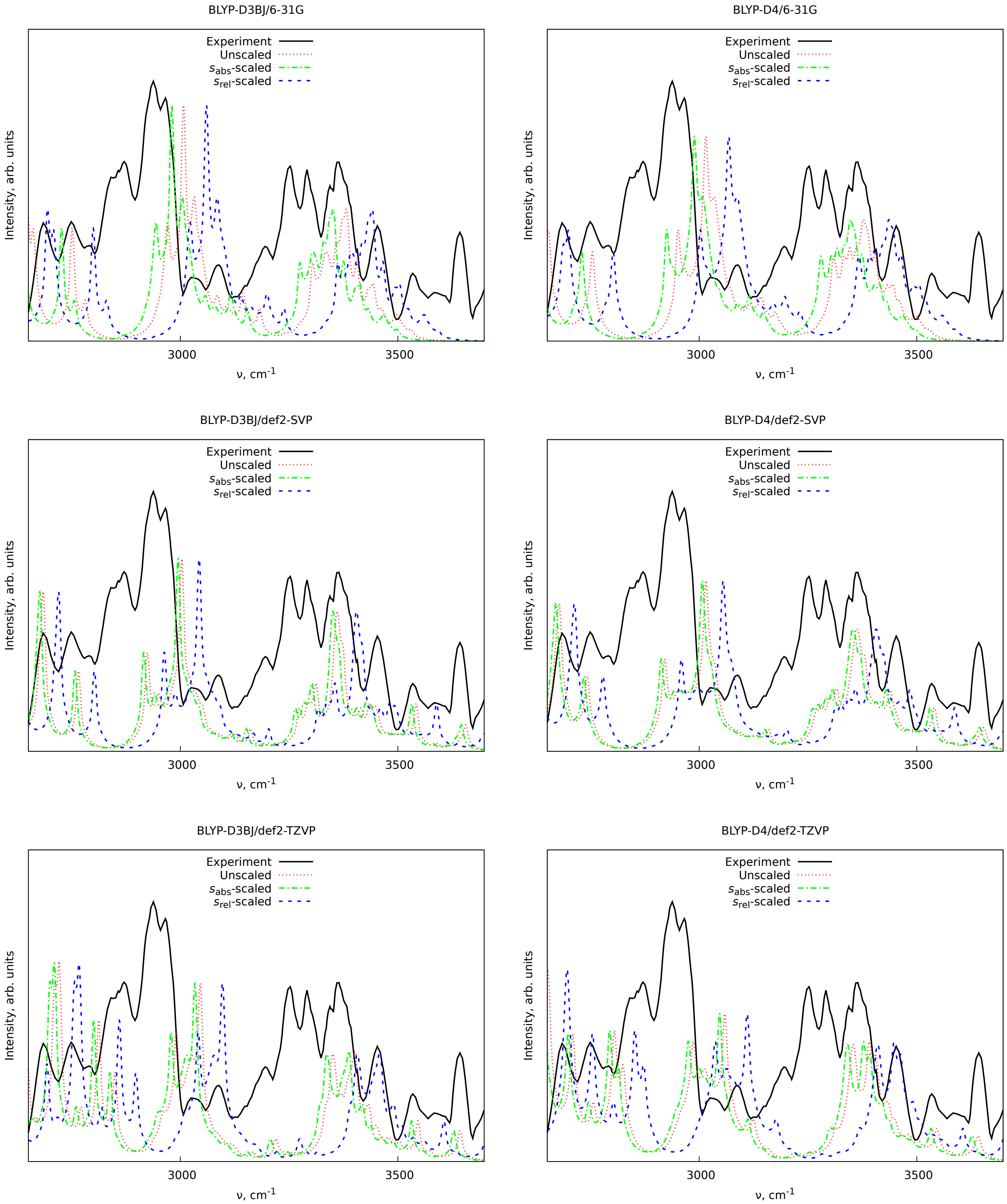

Figure S21 Unscaled and scaled harmonic and experimental (CLIO) spectra of leuenk. Theoretical harmonic spectra were obtained with BLYP-based methods.

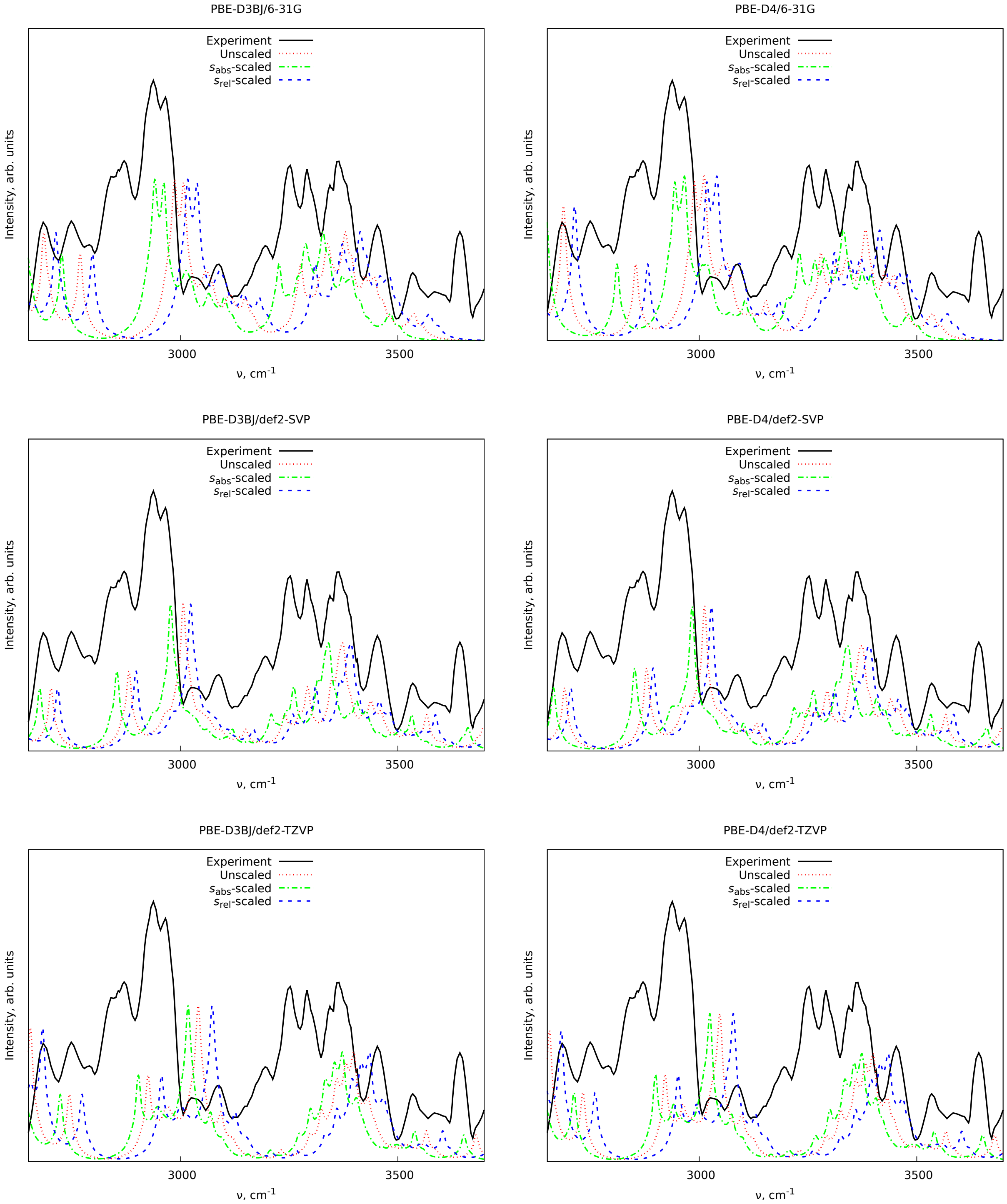

Figure S22 Unscaled and scaled harmonic and experimental (CLIO) spectra of leuenk. Theoretical harmonic spectra were obtained with PBE-based methods.

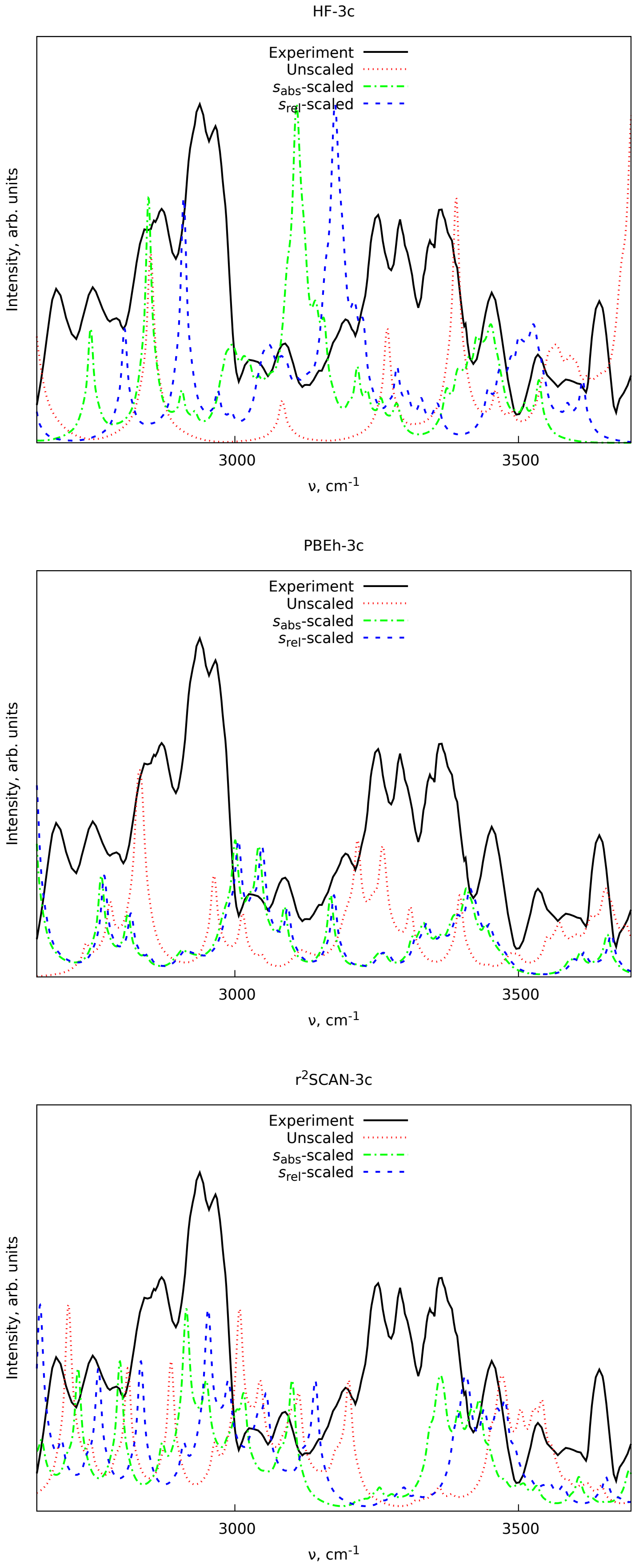

Figure S23 Unscaled and scaled harmonic and experimental (CLIO) spectra of leuenk. Theoretical harmonic spectra were obtained with 3c composite methods.

2.3 Doubly protonated conformer A of gramicidin S

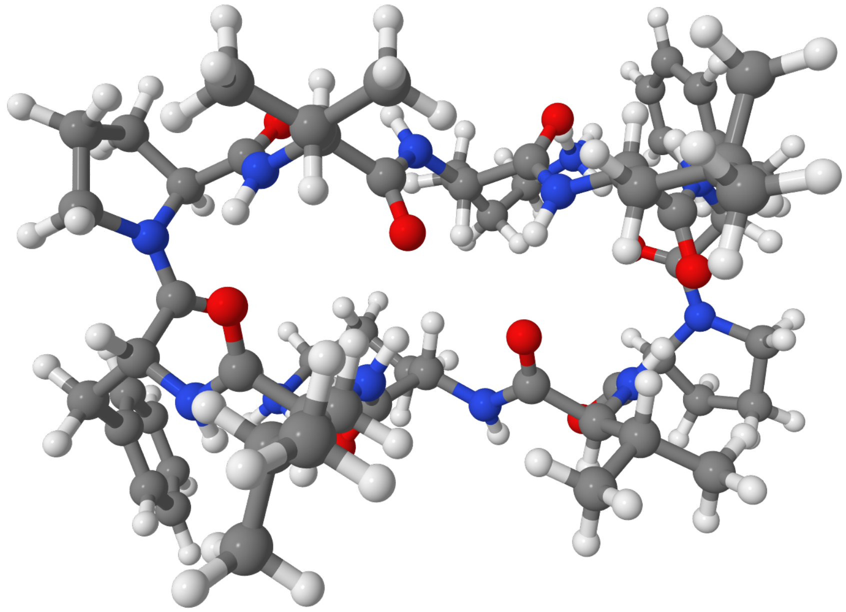

Figure S24 Molecular structure of the doubly protonated gramicidin conformer A.

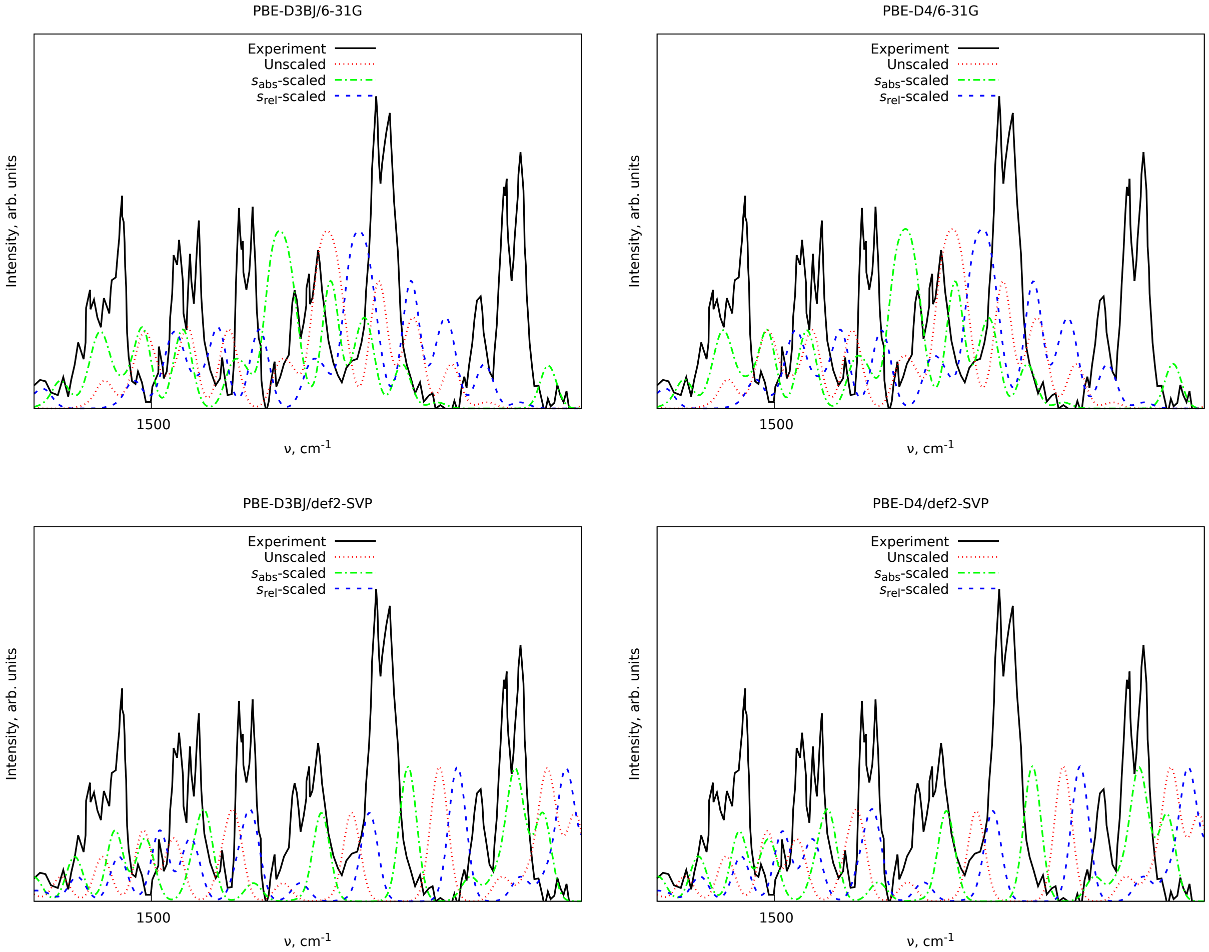

Figure S25 Unscaled and scaled harmonic and experimental spectra of doubly protonated gramicidin conformer A. Theoretical harmonic spectra were obtained with PBE-based methods.

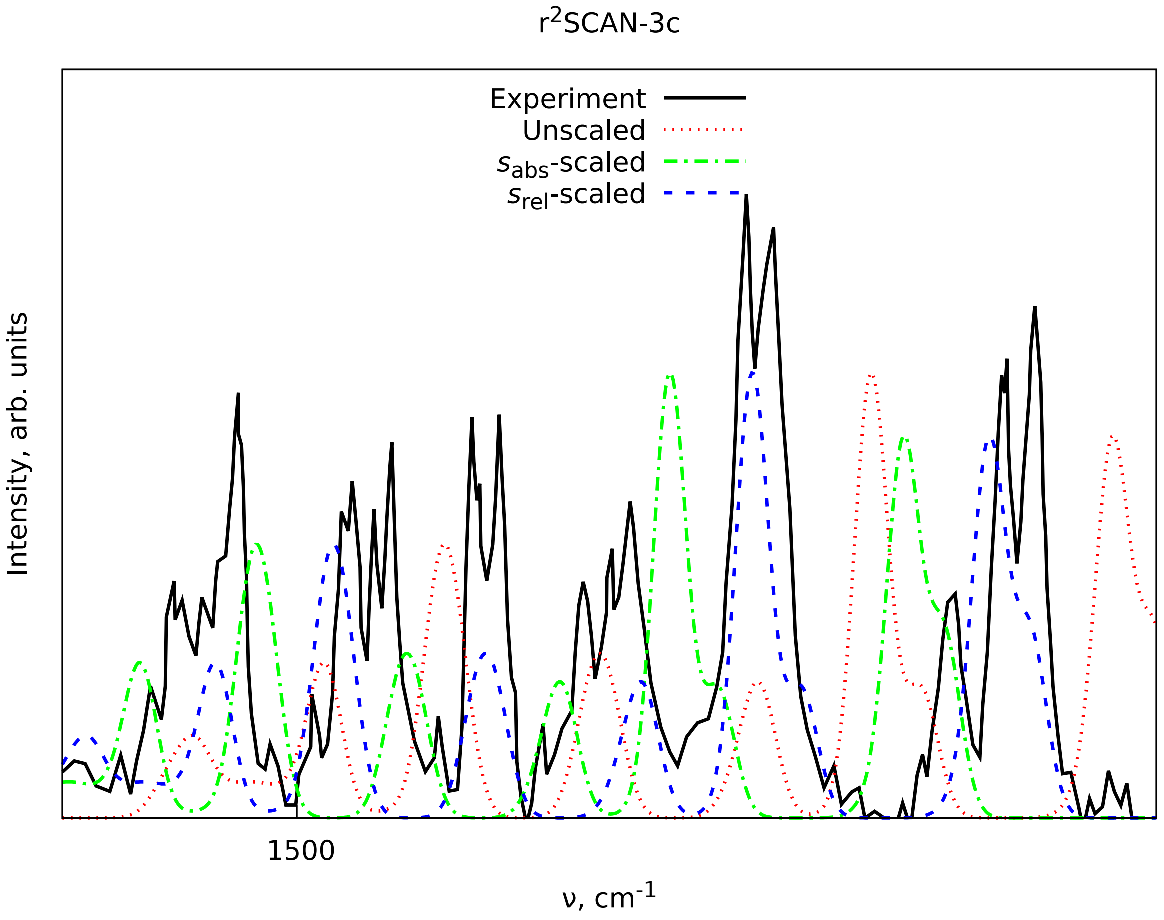

Figure S26 Unscaled and scaled harmonic and experimental spectra of doubly protonated gramicidin conformer A. Theoretical harmonic spectra were obtained with r^{2}SCAN-3c method.
